# *Diaphanous* gene mutation affects spiral cleavage and chirality in snails

**DOI:** 10.1038/srep34809

**Published:** 2016-10-06

**Authors:** Reiko Kuroda, Kohei Fujikura, Masanori Abe, Yuji Hosoiri, Shuichi Asakawa, Miho Shimizu, Shin Umeda, Futaba Ichikawa, Hiromi Takahashi

**Affiliations:** 1 Department of Life Sciences, Graduate School of Arts and Sciences, The University of Tokyo, Meguro-ku, Tokyo, Japan; 2 Department of Biophysics and Biochemistry, Graduate School of Science, The University of Tokyo, Bunkyo-ku, Tokyo, Japan; 3 JST ERATO-SORST Kuroda Chiromorphology Project, Meguro-ku, Tokyo, Japan; 4 Research Institute for Science and Technology, Tokyo University of Science, 2641 Yamazaki, Noda-shi, Chiba, 278-8510, Japan; 5 Department of Applied Biological Science, Graduate School of Science and Technology, Tokyo University of Science, Noda-shi, Chiba, Japan; 6 Department of Molecular Biology, Keio University School of Medicine, Shinjuku-ku, Tokyo, Japan.

## Abstract

L-R (left and right) symmetry breaking during embryogenesis and the establishment of asymmetric body plan are key issues in developmental biology, but the onset including the handedness-determining gene locus still remains unknown. Using pure dextral (DD) and sinistral (dd) strains of the pond snail *Lymnaea stagnalis* as well as its F_2_ through to F_10_ backcrossed lines, the single handedness-determining-gene locus was mapped by genetic linkage analysis, BAC cloning and chromosome walking. We have identified the actin-related diaphanous gene *Lsdia1* as the strongest candidate. Although the cDNA and derived amino acid sequences of the tandemly duplicated *Lsdia1* and *Lsdia2* genes are very similar, we could discriminate the two genes/proteins in our molecular biology experiments. The *Lsdia1* gene of the sinistral strain carries a frameshift mutation that abrogates full-length LsDia1 protein expression. In the dextral strain, it is already translated prior to oviposition. Expression of *Lsdia1* (only in the dextral strain) and *Lsdia2* (in both chirality) decreases after the 1-cell stage, with no asymmetric localization throughout. The evolutionary relationships among body handedness, SD/SI (spiral deformation/spindle inclination) at the third cleavage, and expression of diaphanous proteins are discussed in comparison with three other pond snails (*L. peregra, Physa acuta* and *Indoplanorbis exustus*).

The symmetry breaking event during early embryogenesis is a fundamental step for the correct positioning and morphogenesis of internal organs. As the establishment of left-right (L-R) asymmetry is critical for normal development, the mechanisms governing initiation and maintenance of this asymmetry are tightly regulated and in large part evolutionarily conserved^1^,^2^,^3^,^4^,^5^,^6^,^7^,^8^. Several models have been proposed to account for the initiation, orientation and amplification of the L-R asymmetry. For some vertebrates, symmetry breaking takes place around the L-R organizer, e.g., the node in the mouse, Hensen’s node in chicks, the gastrocoel roof plate in frogs, and Kupffer’s vesicle in fish. In these animals, except for the chick and pig, the fluid flow generated by the rotation of node cilia results in cascades of asymmetric gene expression around the node^2^,^3^,^4^,^5^,^6^,^7^,^8^,^9^. Downstream of leftward flow (so called Nodal flow), the Nodal signaling cascade is activated in the left- but not in the right-lateral plate mesoderm (LPM). Nodal signaling induces the asymmetric expression of Lefty and Pitx2 in the left LPM. Finally, asymmetric Pitx2 acts as a key mediator of leftness^2^,^4^,^6^,^7^,^10^. Similar cascades are conserved among vertebrates and some invertebrates, and provides robustness to L-R asymmetric patterning^3^,^6^,^8^,^11^,^12^,^13^,^14^. The mechanism of L-R establishment, however, seem to vary among deuterostomes^3^,^6^,^7^,^8^.

Evidence has accumulated that L-R body axis in invertebrates is determined through a genetically controlled program. Embryonic handedness choice in *Drosophila* and *Caenorhabditis elegans* involves the unconventional type I myosins and the Gα protein regulating the orientation of the spindle, respectively^14^,^15^,^16^. In addition, some general mechanisms include gap junctions and H^+^/K^+^-ATPase activity as the initial symmetry-breaking steps^3^,^17^,^18^. These processes involve a number of intricately regulated developmental mechanisms, some of which appear to be conserved among deuterostomes.

The freshwater gastropod *Lymnaea (L.) stagnalis* has both the sinistral (recessive) and the dextral (dominant) snails within a species, and its chirality is determined by a single locus that functions maternally at the very early embryonic stage. The unique hereditary systems in *Lymnaea* were described as early as 1923^19^,^20^, but identification of the causative genes and the underlying mechanisms are long-standing unresolved issues. We have shown that the micromanipulation of blastomere rotation at the third cleavage (four- to eight-cell stage) in the direction opposite to the genetically determined sense, reverses the sites of zygotic asymmetric expression of *nodal*–*pitx* genes in later development, resulting in healthy mirror image animals^13^. Further, spiral deformation (SD) and spindle inclination (SI) were uniquely observed only in the dominant dextral embryos particularly during the third cleavage, and they were shown to be strongly linked to the gene that determines the snail chirality^21^,^22^. Based on these results and using pure dextral (DD) and sinistral (dd) strains as well as novel dextral and sinistral lines of F_10_ congenic snails (with 99.9% sinistral- and 0.1% dextral-derived genomes^22^), we here identify the diaphanous-related formin, *Lsdia1,* as the handedness-determining gene and elucidate the mechanisms that establish the L-R body plan in *Lymnaea*. Through quantitative RT-PCR, Northern blotting, whole mount *in situ* hybridization (WISH) and Western blotting, we could discriminate expression of the tandemly repeated highly-conserved *Lsdia1* and *Lsdia2* genes. In sinistral strains, we show that both *Lsdia1* alleles carry a frameshift mutation early in the coding region that leads to protein truncation and loss of full length protein. In the dextral strain, it is already translated prior to oviposition and the gene expression level goes down already at the two-cell stage. No localization of mRNA of *Lsdia1* as well as its tandemly duplicated *Lsdia2* gene was observed during the entire developmental process. We have cloned and sequenced *dia* homologous genes of the pond snails, *L. peregra, Physa acuta* and *Indoplanorbis exustus*, and compared the type of diaphanous proteins, SD/SI at the third cleavage and body handedness.

## Results

### Genetic and physical mapping of the handedness-determining region

**Figure 1:**
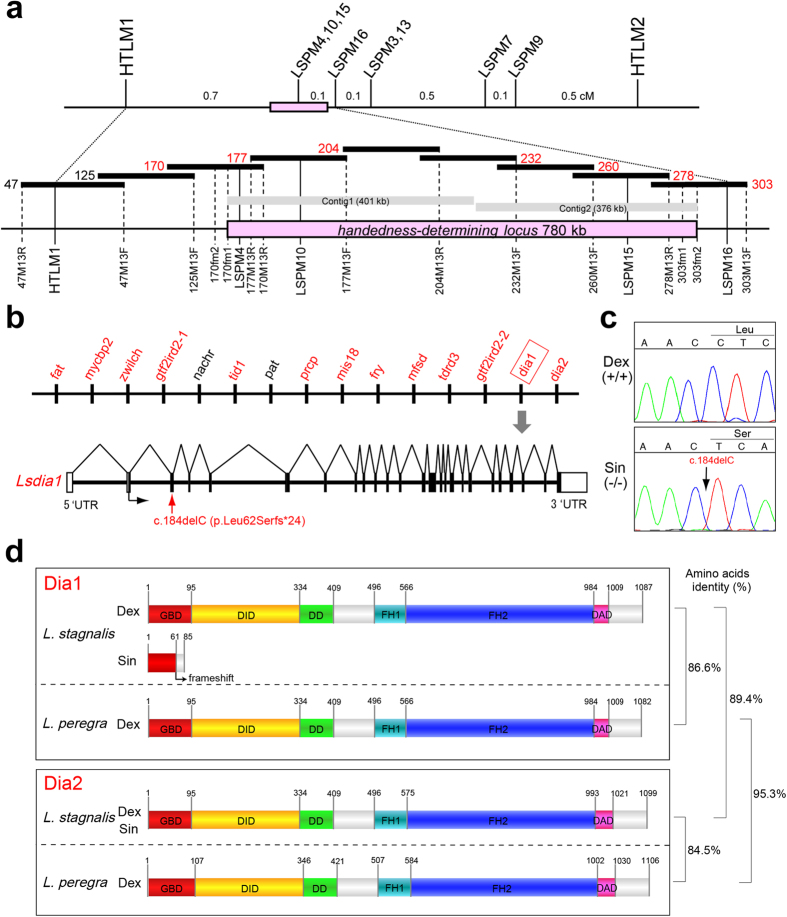
Positional cloning identifies *Lsdia1* as a candidate gene for chirality determination. (**a**) Genetic and physical map of the snail chirality locus. Genetic distances are in cM (1,600 gametes) on the first line of the figure. Closely linked AFLP markers are shown vertically, and black horizontal bars represent BAC clones. Seven BAC clones indicated in red numerals were sequenced and two contigs (Contig 1 and 2) were assembled. There is a small gap (<100 bp) between the two contigs in the current physical maps. (**b**) Upper panel: Schematic representation of 15 candidate genes for snail-handedness determination. Maternally expressed genes are shown in red. Lower panel: *Lsdia1* exon-intron structure and the causative mutation. (**c**) The mutation site responsible for snail chirality is indicated. (**d**) Schematic representation of Dia1 structure of three *Lymnaea* snails (dextral and sinistral *L. stagnalis* and dextral *L. peregra)*, as well as Dia2 of dextral *L. stagnalis* and *L. peregra. Lymnaea* Dia1 contains six conserved domains: Rho GTPase-binding domain (GBD) (IPR010473), diaphanous inhibitory domain (DID), dimerization domain (DD), proline-rich formin homology 1 (FH1) domain, formin homology 2 (FH2) (IPR015425) and diaphanous autoregulatory domain (DAD) (IPR014767). The c.del184C mutation in sinistral *L. stagnalis* generates a premature stop codon (p.Leu62Serfs*24) in the *Lsdia1* mRNA. The amino acid length of Dia1 and Dia2 proteins, and the amino acid sequence homologies are shown at the far right.

As an initial step toward positional cloning, the handedness-determining locus was localized by high resolution genetic mapping to one chromosome. Serial backcrossing experiments were performed in which sinistral hybrids (that had inherited the dextrality determining gene locus from the dextral-derived genome) were backcrossed as a father with the pure sinistral mother^13^,^22^. We genotyped over 1,600 meioses obtained from these crosses, and used amplified fragment length polymorphism (AFLP) typing^23^ to map the handedness-determining locus to a 0.8 cM interval between the AFLP markers HTLM1 and LSPM16. (Fig. 1a; Supplementary Figure S1; Supplementary Tables S1 and S2).

To identify genomic clones that map to the genetic interval containing the handedness-determining gene, we constructed and screened bacterial artificial chromosome (BAC) libraries. Chromosome walking was begun at HTLM1 and seven overlapping BAC clones (BAC clone ID #170, 177, 204, 232, 260, 278 and 303) were identified to span the entire non-recombinant region containing the handedness-determining gene (Fig. 1a).

### *Lsdia1* is the chirality-determining gene

Shotgun sequences of the seven BAC clones were assembled into contigs and placed on the physical map by alignment with AFLP linkage markers (Fig. 1a). Gaps between the adjacent contigs were closed using a primer walking procedure and next-generation sequencing. Then, recombination breakpoints at both ends of the handedness-determining locus were identified by fine mapping, and finally a 780 kb contig was identified with one small gap (<100 bp) (Fig. 1a; Supplementary Table S3). The BAC sequences were annotated for genes using a combination of *ab initio* gene finders (Genscan and FGENESH^24^) and several BLAST algorithms (BLASTN, BLASTX, and TBLASTX). Sixty eight fragmented transcriptional units predicted by *ab initio* modelling were annotated as encoding “hypothetical exons”. Only the genes with transcript evidence and without simple genomic repeats were annotated as candidate genes and on this basis we identified fifteen candidate sequences.

The handedness determining gene is present as a maternal factor in the earliest embryos and thus we determined the expression patterns of all candidate genes in 1-cell embryos from both dextral and sinistral strains. Quantitative real-time PCR analysis revealed that thirteen of the fifteen genes could act with maternal effect (Fig. 1b). Bearing in mind that left-right asymmetry in *Lymnaea* embryos is determined through an actin filament-dependent mechanism^21^, we analyzed five actin-related genes homologous to *fat, mycbp2, fry, dia1* and *dia2* (see Supplement for a description of biological profiles). The full-length cDNA of candidate genes of both dextral and sinistral strains was cloned by RT-PCR, and 5′- and 3′-rapid amplification of cDNA ends (RACE), and their sequences were compared. We identified homozygosity for a single nucleotide deletion in exon 3 of *Lsdia1* from the sinistral strains (accession number: KX387869, KX387870) (Fig. 1b,c), whereas the other genes (accession number: KX387871, KX387872, KX387877- KX387882) did not show any critical mutations that affect evolutionary conserved amino acids within the protein coding sequences (See Methods for Representative candidate genes for snail handedness determination). Thus, *Lsdia1* was the strongest candidate gene for the handedness determination. The 184delC deletion of this gene alters the reading frame, resulting in a frameshift after amino acid 62 and premature termination after amino acid 85 (p.Leu62Serfs*24) (Fig. 1d). While preparing this manuscript for submission, we have noted a publication has just come out that describes the same gene as associated with L-R asymmetry (their *Ldia2* = our *Lsdia1*, Davison *et al*.^25^), however, their results of molecular biology work and hence the conclusions are quite different from ours (see later).

**Figure 2:**
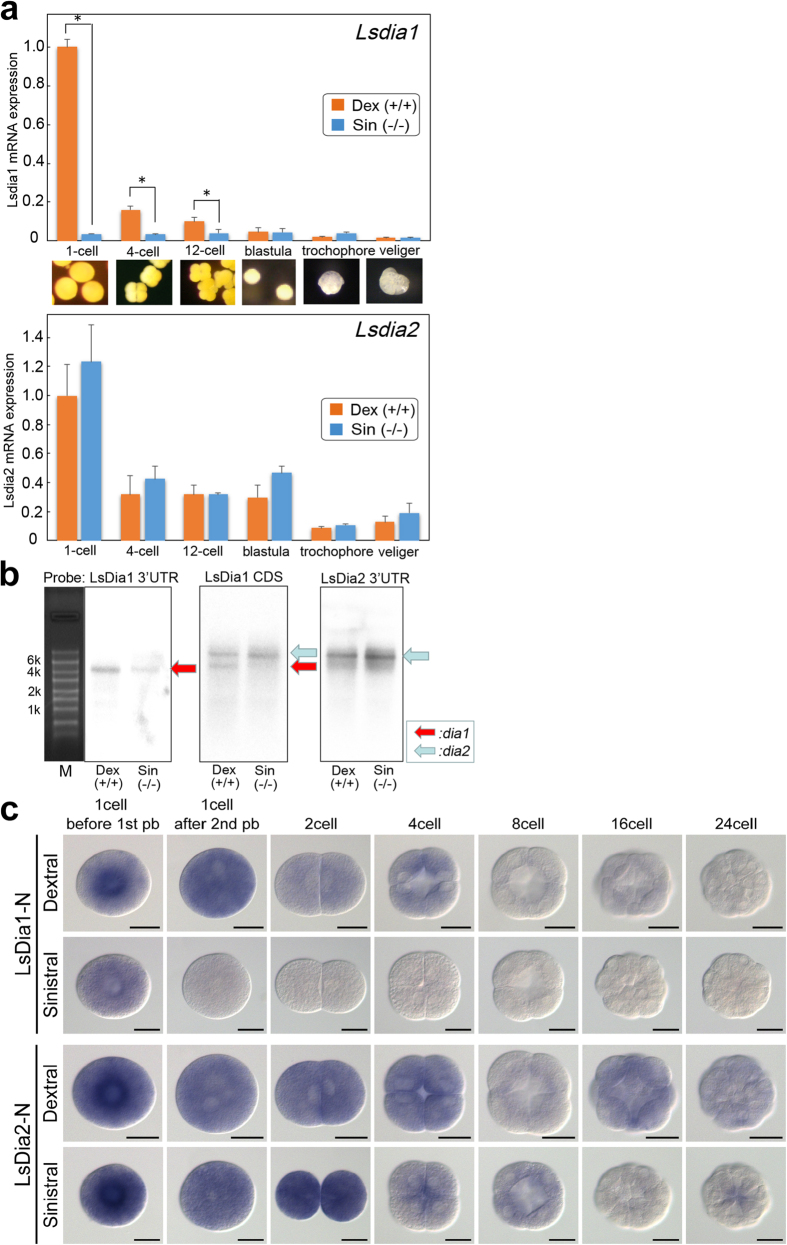
Comparison of *Lsdia* mRNA levels between dextral and sinistral snails during early development. (**a**) Quantitative real time-PCR analysis showing the expression levels of *Lsdia1* and *Lsdia2* mRNA between dextral and sinistral strains. Gene expressions were assessed at six different developmental stages; 1-cell, 4-cell, 12-cell, blastula, trochophore, and veliger with two probes (*Lsdia1*-5′UTR and *Lsdia2*-3′UTR). *Lstubb1* was used as a control. Data are shown as mean ± SD, calculated from four independent experiments. *P < 0.01. (**b**) Northern blot analysis showing the transcript size of *Lsdia1* and *Lsdia2* genes from dextral and sinistral strains. Gene expressions were assessed using 1-cell stage embryos. Three radioactive probes (*Lsdia1*-3′UTR, *Lsdia1*-CDS and *Lsdia2*-3′UTR) were analyzed. *Lsactb* mRNA bands served as a control for monitoring RNA loading. (**c**) Whole mount *in situ* hybridization showing the spatial and temporal expression patterns of *Lsdia1* and *Lsdia2* at seven different embryonic stages; 1-cell before 1st pb extrusion, 1-cell after the 2nd pb extrusion, 2-cell, 4-cell, 8-cell, 16-cell, and 24-cell stages. Gene expressions were assessed with a probe made for the CDS N-terminus region of *Lsdia1* which overlaps the probe region for the northern blotting, and the CDS N-terminus region of *Lsdia2*. All images are animal pole views. Scale bar indicates 50 μm.

LsDia1 is characterized by the presence of conserved domains of diaphanous-related formin, but the frameshift mutation in sinistral snails leads to the complete loss of all these functional domains (Fig. 1d). LsDia2 shows 89% amino acid sequence similarity to LsDia1, but we could not detect any differences in LsDia2 between dextral and sinistral strains at the amino acid sequence (accession number: KX387871, KX387872) and expression level (see Fig. 2c and Supplement Figure S2). The diaphanous formin is a conserved functional gene that catalyzes actin nucleation and polymerization to form actin filaments^26^,^27^,^28^. It can also stabilize microtubules and bind to Rho GTPases and formins. The gene matches with our previous findings of a direct link of the handedness-determining gene to SD and SI at the third cleavage, as well as the finding that SD precedes SI with a cross talk between them, as nocodazole treatment of *L. stagnalis* embryos causes even more pronounced SD without spindle formation^21^.

### *Lsdia1* is hardly expressed in the sinistral strain throughout development - temporal expression patterns of *Lsdia1 and Lsdia2*

As *Lsdia1* is the strongest candidate gene for the handedness determination in *L. stagnalis*, we carried out detailed real-time PCR experiments for the quantitative detection of RNA transcript of *Lsdia1* and *Lsdia2* in the sinistral and dextral strains of *L. stagnalis* during the embryogenesis. cDNA base sequences of *Lsdia1* and *Lsdia2* are very similar (accession number: KX387869- KX387872), however, we were able to make real-time PCR primers which discriminate *Lsdia1* and *Lsdia2* by targeting the 5′ UTR of *Lsdia1* and 3′ UTR of *Lsdia2* (86 nt and 139 nt, respectively. nt = nucleotides) (Fig. 2a). The results clearly show that *Lsdia1* is hardly expressed in the sinistral strain for the entire period of embryogenesis, i.e. from the 1-cell stage to the veliger stage. In contrast, substantial levels of *Lsdia1* mRNA were found in the dextral strain at the 1-cell stage (dex/sin ratio: 40.4, *P* < 0.01), however, it decreased sharply through the developmental stages (Fig. 2a top). Only ca. 1/6 amount of *Lsdia1* mRNA is present at the 4-cell stage as compared with the 1-cell stage, and it is close to the background level at the veliger stage (Fig. 2a top).

*Lsdia2* is expressed in both the dextral and the sinistral strains at the 1-cell stage, and the level decreases gradually during the early stage of development in both handedness (Fig. 2a bottom). All the data are shown as mean ± standard deviation, calculated from four independent experiments.

The expression level of *Lsdia1* was also assessed in both dextral and sinistral strains by hybridization of probes to northern blots. The probes were made to the 3′ UTR of *Lsdia1* (366 nt) and *Lsdia2* (558 nt) to distinguish between *Lsdia1* and *Lsdia2*. A probe to the *N*-terminus region of *Lsdia1* CDS (273 nt) was also made. The RNA samples were derived from the 1-cell stage embryos from both strains. With the probe made for the 3′UTR region of *Lsdia1*, expression of only *Lsdia1* (and not *Lsdia2*) was detected, whose signal was much stronger in dex (+/+) than in sin (−/−) (Fig. 2b left). Northern blotting with probe made to hybridize the 3′ UTR region of *Lsdia2* showed a strong band for *Lsdia2* for both dex (+/+) and sin (−/−) (Fig. 2b right). In contrast, the probe made for the *N*-terminal region of *Lsdia1* CDS recognizes both *Lsdia1* and *Lsdia2*, and has exhibited two bands corresponding to *Lsdia1* and *Lsdia2* for dex (+/+), but only *Lsdia2* band for sin (−/−) (Fig. 2b middle). These results agree well with the real-time PCR results (Fig. 2a).

### No localization of *Lsdia1 and Lsdia2* - Spatial expression patterns of *Lsdia1 and Lsdia2*

We carried out WISH (whole mount *in situ* hybridization) experiments to reveal the temporal and spatial expression of *Lsdia* mRNAs during early embryogenesis, i.e., 1-cell before the first polar body (pb) extrusion, 1-cell after the second pb extrusion, 2-cell, 4-cell, 8-cell, 16-cell and 24-cell stages for the sinistral (−/−) and the dextral (+/+) strains (Fig. 2c and Supplement Figure 2). Short length anti-sense *Lsdia1* probes used for the northern experiments (366 nt *Lsdia1* 3′ UTR probe and 273 nt *Lsdia1* CDS probe) did not reveal expression of *Lsdia* genes even with longer hybridization and staining. This may be because the short probes cannot withstand extensive washing processes required to achieve high selectivity in the WISH experiments. To detect low-level expression (as judged from our real-time PCR experiments) with high selectivity, longer probes are required, although this may increase the chance of cross hybridization with other mRNAs of similar sequences. An anti-sense *Lsdia1* probe of 1002 nt was made corresponding to the N-terminus region of CDS (LsDia1-N), and, in fact, the probe selectively detected *Lsdia1* expression. We believe this is due to the extensive post-hybridization RNaseA treatment which was performed to remove non-specific hybridization products. Similarly, a 1002 nt anti-sense *Lsdia2* probe was made to the N-terminus region of LsDia2 CDS (LsDia2-N), which also seems to detect *Lsdia2* expression selectively. The LsDia1-N probe for WISH experiments covers the region of LsDia1 CDS probe (273 nt) used for the northern blotting. WISH analyses using respective sense probes and that for maternal β-tubulin and actin were carried out as control to check the integrity of our WISH experiments. The results showed only the background level expressions (Supplementary Figures S2 and S3). With LsDia1-N probe, WISH experiments show prominent expression only at the 1-cell stage prior to first pb extrusion for the sin (−/−) strain. After this stage, the *Lsdia1* expression drops sharply close to the background level already at the 1-cell stage after second pb extrusion in the sin (−/−) (Fig. 2c). In the case of dex (+/+), strong *Lsdia1* expression is observed at the 1-cell stage and the expression level decreases gradually towards the 8 -cell stage, and becomes almost the background level at the 24-cell stage (Fig. 2c). With LsDia2-N probe, *Lsdia2* expression was observed both in dex (+/+) and sin (−/−) embryos with similar time course change to each other, i.e., strong at the 1-cell and 2-cell stages and decreases substantially at the 4-cell stage, and then gradually towards the 24-cell stage. Slight increase of the expression level of *Lsdia2* of dex (+/+) at the 16-cell stage may reflect the zygotic expression of the gene which starts about this stage. These results agree well with our real-time PCR experiments (Fig. 2a).

Remarkably, no localization of the expression of *Lsdia1* nor *Lsdia2* was observed within a cell at the 1-cell stage, nor among blastomeres in the 2- or 4-cell stages (Fig. 2c). The results are in striking difference from the recently reported WISH results of the same gene [our *Lsdia1* = *Ldia2* of Davison *et al*.^25^]. They did not discriminate *Lsdia1* and *Lsdia2* gene expressions, and reported strong asymmetrically localized expression confined to one of the blastomeres or a part of a blastomere at the 2- and 4-cell stage embryos. Some of their WISH results display similar localization even for *Lfry* (= our *Lsfry*) as well at the 2-cell and 4-cell stages^25^. They fixed embryos while in their capsules- a viscous environment rich in various chemicals - whereas we cultured embryos in their capsules until they reached respective developmental stages, and then took them out of the capsules for fixing and hybridization as suggested in earlier studies^29^. It is well documented and confirmed here that decapsulation has no mal effects on subsequent embryonic development^29^.

We have carried out WISH experiments carefully. All the embryos at different developmental stages were treated in one well under the same conditions including the staining duration, for respective LsDia1-N and LsDia2-N probes. At least two independent experiments were carried out (Fig. 2c, Supplementary Figure S2). Extension of staining duration did not reveal any localized expression. In all these experiments, we did not observe any asymmetric localization of *Lsdia1* and *Lsdia2* mRNAs.

### Protein studies - LsDia1-specific antibody and developmental expression of LsDia1

**Figure 3:**
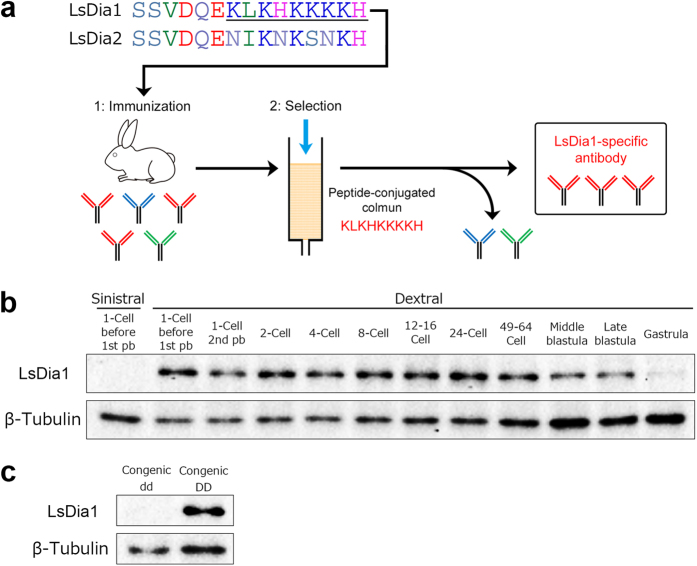
Generation of an LsDia1-specific antibody and developmental expression of LsDia1 protein. (**a**) Comparison of the amino acid sequences of the C-terminal region between LsDia1 (residues 1054–1068) and LsDia2 (residues 1066–1080), which were selected as a target site for making anti-LsDia1 antibody. Underlined sequence indicates the recognition site of anti-LsDia1 polyclonal antibodies. (**b**) Western blotting analyses showed that LsDia1 protein is present in the dextral embryos from 1-cell immediately after oviposition to the blastula stage, but is not detectable for the sinistral embryos even at the 1-cell stage prior to 1st pb extrusion. LsDia1 and β-Tubulin levels were analyzed sequentially using the same blot. The whole images of blot showing the single band of correct MW for LsDia1 and β-Tubulin, respectively, are presented in Supplementary Figure S4. (**c**) Presence and absence of LsDia1 protein in early stage embryos of the congenic (F10) dd strain (1–4-cell) and DD strain (1-cell), respectively.

The amino acid sequences of LsDia1 and LsDia2 are very similar, but we focused on the C-terminal region where there is a slight difference in the amino acids between these two proteins, i.e., C-terminal region of LsDia1 (SSVDQEKLKHKKKKH, residues 1054–1068) and LsDia2 (SSVDQENIKNKSNKH, residues 1066–1080) (Fig. 3a). Anti-LsDia1 antibody was produced by immunizing rabbits with the peptide for LsDia1, and further selected by affinity column conjugated to the synthetic peptide KLKHKKKKH (Fig. 3a, underlined). The western blot analyses show a complete absence of LsDia1 protein in sin (−/−), whereas for dex (+/+), the protein is present from oviposition to the gastrulation stage (Fig. 3b, and the whole blot in Supplementary Figure S4). The absence of LsDia1 in sin (−/−) and presence in dex (+/+) is consistent with our real-time PCR, northern blotting and WISH results for the mRNA. The subcellular localization pattern has been analyzed by whole mount immunofluorescent staining in fixed early cleaving embryos, however, the specific localization signals have not been detected so far. The epitope sequence selected for making anti-LsDia1 antibody might not be suitable for the whole mount immunostaining. Further work with new methodology is required. Interestingly, although the mRNA expression of *Lsdia1* in dex (+/+) drops substantially after the 1-cell stage, the western blotting clearly shows the persistence of LsDia1 protein through to gastrulation. The protein is already present before the first pb extrusion - indicating the gene is translated in the oocyte- and, prior to the third cleavage allowing preparation for the critical third cleavage, consistent with the observation of chiral twisting of embryos at the first cleavage^30^.

We have succeeded in constructing the lines of congenic strains F_10_ that on average inherit 99.9% of the sinistral-derived genome and only 0.1% of the genome derived from the dextral strain^22^. Our western blotting clearly shows no detection of LsDia1 protein in F_10_-congenic dd (1–4 cell stages) but substantial amount in F_10_-congenic DD (1-cell) (Fig. 3c) revealing that *Lsdia1* is the strongest candidate gene for the chirality determination.

### Type of Dia proteins and SD/SI in pond snails

**Figure 4:**
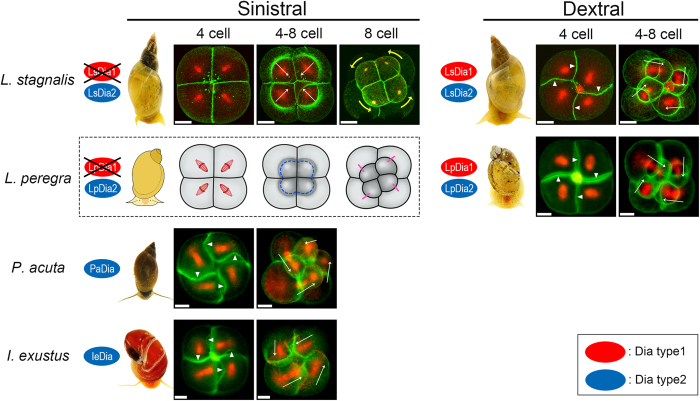
Dia gene types and SD/SI at the third cleavages for the three pond snails studied. Fixed embryos during the third cleavage were stained for actin filament (green) and *β*-tubulin (red). White arrowheads and arrows indicate SD and orientation of spindles, respectively. Yellow arrows indicate the rotating direction of small blastomeres towards the 8-cell stage of sinistral *L. stagnalis* embryo. All were observed from the animal pole side. Scale bar: 20 μm. Sinistral only snails, *P. acuta* and *I. exustus*, possess only Dia2 type proteins (blue) (PaDia and IeDia), and exhibit counter-clockwise SD and SI. Snails which belong to *Lymnaeidae, L. stagnalis* and *L. peregra* have both dominant dextral and recessive sinistral animals in the wild. The dominant dextral ones of the two species have both Dia type 1 (red) and Dia type 2 (blue) proteins and exhibit clockwise SD and SI, whereas the recessive sinistral *L. stagnalis* possesses only Dia type 2 (blue) protein showing no SD/SI. Figures for *L. stagnalis* were adapted from Shibazaki *et al*.^21^. Lack of SD/SI was observed for sinistral *L. peregra*, but no photographs of the adult snail nor embryo images are available, and hence are shown schematically. (Sinistral *L. peregra* is now unavailable as a laboratory strain). The photographs of snails were taken by ourselves.

We have previously reported the direct link of the handedness-determining gene to SD and SI at the third cleavage^13^,^21^,^22^. To compare dia gene function in pond snails of differing chiralities, we have cloned and sequenced *dia* homologous genes in *L. peregra (Lp*), *Physa acuta (Pa*) and *Indoplanorbis exustus (Ie*), and compared the relationships between their body handedness, the presence and absence of Dia type1 and/or Dia type2 proteins, and the presence and absence of SD/SI at the third cleavage (Fig. 4; Supplementary Figure S5).

The differences between LsDia1 and LsDia2 are confined only to the numbers of proline repeat in the FH1 domain and amino acid sequence at the C-terminus domain. Sinistral only snails, *P. acuta* and *I. exstus* possess only Dia type2 proteins (PaDia and IeDia, respectively; accession number: KX387875 and KX387876), and exhibit counter-clockwise SD and SI. Snails which belong to *Lymnaeidae*, i.e., *L. stagnalis* and *L. peregra*, have both dominant dextral and recessive sinistral animals in the wild. The dominant dex (+/+) of both species have Dia type1 as well as Dia type2 proteins (Fig. 1d, LpDia1 and LpDia2; accession number: KX387873 and KX387874) and exhibit clockwise SD and SI, whereas the recessive *L. stagnalis* sin (−/−) possesses only Dia type2 protein and is devoid of SD/SI (Fig. 4). Sinistral *L. peregra* also showed counterclockwise micromere rotation without SD/SI, but the last remaining strain worldwide was unfortunately lost before proper records of the embryo images including the actin/tubulin staining could be made.

### Gene functional analyses

We examined the effects of drug inhibition using SMIFH2, an FH2 domain inhibitor^31^ of Dia proteins for both dex (+/+) and sin (−/−) embryos of *L. stagnalis* carried out using freshly oviposited eggs. If the drug concentration was more than 10 μM, all the dex (+/+) embryos died. At 7.5 μM, only 5 out of 63 embryos developed to the 4-cell stage and development was arrested. No inhibition was observed at 2.5 μM. At 5.0 μM concentration, 30 out of 109 embryos developed to the 8-cell stage of which only 13 developed further. Those which survived the drug treatment showed SD/SI and clockwise rotation as usual. Other embryos whose development stopped exhibited faulty spindle orientation, blastomere shape and the timing in micromere formation, and hence SD/SI could not be defined. For sin (−/−) embryos, at 5.0 μM level, 32 of 113 embryos developed to the eight-cell stage, out of which 26 developed further. The surviving embryos exhibited anti-clockwise micromere rotation without SD/SI as for the normal untreated ones, and those whose development stopped showed substantial deformation. Given the lethality of the drug, we concluded that the results do not truly reveal the function of Dia proteins controlling the chirality of spiral cleavage. Davison *et al*. reported inhibition experiments using the same drug although the concentration was as high as 100 μM, and the drug was added shortly after the completion of the second cleavage. They observed that a very small number of surviving embryos behaved like sinistral as they lost SD/SI, but that was followed by dextral type clockwise rotation^25^. Thus, it is difficult to accept that “the anti-formin drug treatment converts dextral snail embryos to a sinistral phenocopy”.

Injection of *in vitro* synthesized *Lsdia1* mRNA into the 1-cell stage sinistral embryos did not induce SD/SI nor clockwise rotation at the third cleavage. Injection of the antisense morpholino oligonucleotides to the dextral 1-cell embryos did not induce sinistrality. This is most likely because LsDia1 is present as protein already immediately after the oviposition, as shown by western blot analyses (Fig. 3b). Hence it is too late to inhibit the mRNA translation or to rescue the gene’s function at this stage.

We found that a systemic RNAi response was triggered when dsRNA condensed by the polycation polyethylenimine (PEI) was injected into the body cavity of the adult *L. stagnalis*. However the RNAi effect was not transferred to offspring transovarially and chiral reversal was not confirmed. To prove that *Lsdia1* is the century sought after handedness-determining gene, gene function analyses by molecular biology methods such as gene editing will be desirable. In preliminary experiments, we have shown that gene editing of *Lsdia1* in the dextral *L. stagnalis* produced offspring that lacked SD/SI followed by counterclockwise rotation of the micromeres, behaving exactly like the wild-type sinistral *L. stagnalis* i.e. fully consistent with *LsDia1* being the chirality-determining gene in this organism. Further extensive work following the effects on the next generations should provide vital information on chiromorphogenesis.

## Discussion

Previously we have shown that the handedness of *L. stagnalis* can be switched by mechanical micromanipulation of the embryos at the third cleavage, i.e., sinistralization of dextral and dextralization of sinistral embryos. The manipulated embryos grew to be healthy, fertile individuals exhibiting complete *situs inversus*, but their offspring’s handedness reverted to that dictated by their genotype as expected^13^. We have shown that the manipulation transfers the zygotic expression of *nodal* and *pitx* to the mirror image position. Unlike the mouse which expresses *Nodal* asymmetrically at 8.0–8.5 days for a short period of time^32^, *L. stagnalis* starts the expression during the 32–64 cell stages in the micromeres originated from the C macromere^13^, and the expression continues up to the veliger stage and then decreases gradually and eventually disappears when the asymmetrical morphology becomes noticeable and the shell starts to form^33^. In the equal cleaving *gastropods* such as *L. stagnalis*, all four cells are of equal size at the 4-cell stage. The cell pairs A/C and B/D can be distinguished from their appearance, but A and C are indistinguishable, as are B and D^33^. Our micromanipulation at the third cleavage does not move the macromere C nor micromere 1c to the other side of midline, however, it shifts the *nodal* expression to the mirror-image site^13^. Thus, our experiments have clearly shown that the cell fate of A or C is not yet specified at the 4-cell stage^33^. These facts indicate that handedness determination is being prepared from the 1-cell stage but is not yet fully established prior to the third cleavage, so that it can be easily overridden. We have already shown that the similar reversing manipulation at the first and the second cleavage loses the effect at the third cleavage resulting in the reversion to the original chirality^13^. These results do not match the recent report on asymmetric gene expression at the 2- and 4-cell stages of *L. stagnalis* embryos indicating that individual macromeres have an identity at the 2-cell stage^25^. Our results of no localization and very low level of *Lsdia1* mRNA agrees well with our finding of cell-fate determination at the third cleavage based on our mechanogenetics experiments, as well as with models previously proposed^34^,^35^,^36^. In the unequal cleaving gastropods such as *Ilyanassa*, the D blastomere is much larger than the other A, B, and C cells at the 4-cell stage and thus can be easily recognized. We are currently working to find the beginning of cell-fate determination at the molecular level.

SD/SI are important for embryos to be robust. Lack of SD/SI as in wild sinistral *L. stagnalis* sometimes results in insufficient subsequent micromere rotation such as partial-anti-clockwise, neutral or partial clockwise rotation, even when the eggs are contained inside the capsules. Most of those embryos which do not show proper anti-clockwise rotation die during the developmental processes^22^.

If we take account only of the *L. stagnalis* results, LsDia1 appears to be responsible for the SD/SI and the right-handed micromere rotation. However, sinistral snails *P. acuta* and *I. exustus* with only Dia type 2 exhibit counterclockwise SD/SI and leftward micromere rotation. Thus, interestingly, in these snail species, Dia type 2 protein appears to be responsible for the actin cytoskeletal dynamics causing SD/SI.

We could create healthy dextral snails of *P. acuta* by mechanical manipulation^37^, showing that L-R symmetry can be switched at the third cleavage even for sinistral only snails expressing only Dia type 2. Together with the successful mechanical chirality reversal from left to right and right to left for *L. stagnalis*^13^ regardless of the presence or absence of Dia type 1 protein, it can be hypothesized that Dia type 2 has a role of leading the embryos towards the activation of Nodal signaling pathway based on the macromere-micromere contacting geometry which is established at the 8-cell stage, regardless of whether that is established genetically or artificially. This must be achieved by rearranging the cell positions particularly that of the organizer macromere 3D at the 24-cell stage, activating MAPK within the 3D macromere^38^,^39^, and then eventually activating the *nodal* gene by MAPK.

Dia type 1 and 2 are tandemly repeated (Fig. 1b), and one of them is presumably derived from the other during evolution. Thus, the proteins must have overlapping roles as the lack of LsDia1 is not lethal, but at the same time, they likely have some specialized functions. Our preliminary impairing experiments of the *Lsdia1* gene in the dex (+/+) embryos by gene editing techniques appear to create wild-type sinistral snails (to be published). LsDia1 is translated already at the oviposition in the dex (+/+) embryos which means it must have an important role in the cytoskeletal dynamics at the very early development. Moreover, the protein persists through to gastrulation although the mRNA level goes down sharply during the early development (Fig. 3b, Supplementary Figure S4).

The direction of SD/SI can be either right-handed or left-handed. *L. stagnails* with only Dia type 2 does not produce SD/SI. How to explain these observations? One hypothesis is that possession of both Dia type1 and type2 proteins is required to be dextral, and that sinistral-only snails with Dia type 2 such as *P. acuta* and *I. exustus* might have existed first, whose chirality may be related to the intrinsic asymmetry associated with self-organization of actin cytoskeleton^40^,^41^. The addition of a tandemly repeated *Dia type1* gene to the original *LsDia2* somehow created *Lymnaeidae* snails with dextral body plan (also stated in Davison *et al*.^25^), and as a consequence of the gene repetition, the function of making SD/SI in LsDia2 or LpDia2 might have been transferred to LsDia1 or LpDia1 in *Lymnaeidae.* Hence, the loss of LsDia1 or LpDia1 which happened later might have led to the loss of SD/SI, a disadvantage for their survival, which makes the sinistral strains prone to extinction^22^. This interpretation suggests that the direction of rotation and the style of cleavages, i.e., with and without SD/SI may be independent events. Clearly, more work is needed to define precisely the role of the Dia type 1 and/or Dia type 2 proteins in the coiling of different snail species.

There are still so many questions to be answered regarding the mechanism of chiromorphogenesis. How is dextrality achieved by the presence of both LsDia1 and LsDia2? How do Dia proteins cause SD at the molecular level? What determines the direction of rotation? It appears that new findings reveal interesting and important facts but at the same time deepen the mystery regarding L-R body asymmetry formation. Where snails have only one type of *dia* gene, tampering with the gene is lethal. Thus, *Lymnaeidae,* especially *L. stagnalis* which has animals of both chirality in the wild and possesses two slightly different copies of Dia proteins, is an ideal and tractable system for study. Gene editing experiments as well as spatial and temporal distribution studies of LsDia proteins during embryogenesis will certainly give insights into the role of these proteins in the still mysterious handedness-determining steps of Lophotrochozoa.

## Methods

### Construction of congenic strains of *L. stagnalis*

Left-right asymmetry in *L. stagnalis* is determined by a single locus that functions maternally at the early developmental stage, and dextral coiling is dominant over sinistral^42^,^43^. The manner of inheritance in *L. stagnalis* is basically the same as that in related species *L. peregra*^19^,^20^,^44^. We have carried out backcross breeding between dextral and sinistral inbred strains of *L. stagnalis* and constructed congenic strains in which only the chirality locus was introduced to the sinistral genetic background^22^. The backcross progeny obtained was typed for its genotype and the phenotype of the next generation offspring oviposited by it. Following 10 generations of backcrossing, the strains (F10) were considered fully congenic, theoretically 99.9% identical to the sinistral strains at all loci except those linked to the chirality locus of interest^22^. These long-term crossings and genetic linkage analyses made it possible to narrow down the target locus.

The next generation of F10 congenic snails with F_11_DD and F_11_dd genotype were established from one of the dextral F_10_ congenic snails (F_10_Dd) by self-fertilization, and then both dextral and sinistral F10-congenic strains were maintained by either self-fertilization or sibling mating. We have two congenic strains, BC1 and BC2, which have different parental snails for backcrossing. To date, the F10-congenic strains reached F22-F23 and F25-F26 generations for BC1 and BC2, respectively. Similarly, the dextral and sinistral pure strains were established by self-fertilization until F10 and F15 for dextral and sinistral strains, respectively, and then maintained by either self-fertilization or sibling mating. They have now reached up to F28-F29 and F26-F27 generations for the dextral and sinistral pure strains, respectively^13^,^22^.

### Mapping of the handedness-determining locus in *L. stagnalis*

In any generation through the continuous backcross experiments, a large number of the congenic individuals were obtained by increasing the pairs of parents. Screening of molecular markers linked to the genotype of shell chirality was performed by using the population to map the handedness-determining gene to a single locus. The genotype, either dextral (Dd) or sinistral (dd), was identified by confirming the shell chirality of offspring^22^.

The molecular markers were developed on the basis of amplified fragment length polymorphism (AFLP)^23^. This technique does not require any genomic information in advance, thus it was suitable for making linkage markers in *L. stagnalis*, for which the complete genomic sequence has yet to be determined. AFLP protocols followed the method described by Vos^23^. The 5′-terminal end of the selective-amplification primer was labeled with a fluorescent substance (FAM), and fragments amplified by using the primer were analyzed on an ABI3130xl Genetic Analyzer with GeneMapper software v3.7^45^.

Genomic DNA was extracted from liver or foot tissue collected by dissection after confirming the shell chirality of the progeny or from a piece of foot tissue resected from an individual whose genotype was confirmed later. Screening by AFLP typing was performed for a total of 1,600 panels obtained by BC1 strains (F2, 383; F8, 207; F9, 403) and BC2 strains (F6, 364; F7, 243) (Supplementary Table S1).

In the panel screening by AFLP typing, *Eco*RI/*Mse*I-AFLP linkage markers HTLM1 and HTLM2 were found, which were located so as to sandwich the handedness-determining locus on the dextral derived genome (Fig. 1a). Both HTLM1 and HTLM2 were amplified from the pure dextral strains (DD) and the dextral congenic snails (Dd), but these were not amplified from the pure sinistral strains (dd) and the sinistral congenic snails (dd) in panel screening (Supplementary Figure S1a). Snails with only one of the two markers sometimes appeared, and those were identified as recombinant individuals. Eleven and twenty snails were obtained that had produced recombination at the HTML1 or HTLM2 side, respectively (Supplementary Table S1). Individuals with both markers did not produce a sinistral offspring, nor individuals lacking both markers produced a dextral offspring. Thus, it was assumed that a double-crossover between HTLM1 and HTLM2 did not occur. The genotype of snails which died before laying was presumed from the pattern of AFLP markers.

In order to identify the precise recombination points between HTLM1 and HTLM2, a search for linkage markers by *Pst*I/*Mse*I-AFLP^46^ was performed by using the recombinant individuals obtained by *Eco*RI/*Mse*I-AFLP. As a result, eight molecular markers (LSPM3, 4, 7, 9, 10, 13, 15, 16) were found in the region (Supplementary Figure S1b, Supplementary Table S2). In particular, LSPM4, 10, and 15 were completely linked to the dominant handedness-determining gene, and their positions in the non-recombination region were identified by the physical mapping (Fig. 1a). This approach traced the handedness-determining locus allowing the single gene to be identified by genetic mapping.

Genetic distances between the neighboring markers were expressed using the kosambi mapping functions. The primers used for selective-amplification of AFLP markers were as follows: HTLM1 (EcoRI+AGT: FAM-5′-GACTGCGTACCAATTC AGT-3′, and MseI+GTG: 5′-GATGAGTCCTGAGTAA GTG-3′, marker fragment length 206 bp); HTLM2 (EcoRI+AAG: FAM-5′-GACTGCGTACCAATTC AAG-3′, and MseI+ACT: 5′-GATGAGTCCTGAGTAA ACT-3′, marker fragment length 176 bp); LSPM4 (PstI+AC: FAM-5′-GACTGCGTACATGCAG AC-3′, and MseI+CAT: 5′-GATGAGTCCTGAGTAA CAT-3′, marker fragment length 281 bp); LSPM10 (PstI+GT: FAM-5′-GACTGCGTACATGCAG GT-3′, and MseI+CGT: 5′-GATGAGTCCTGAGTAA CGT-3′, marker fragment length 187 bp); LSPM15 (PstI+CG: FAM-5′-GACTGCGTACATGCAG CG-3′, and MseI+CCC: 5′-GATGAGTCCTGAGTAA CCC-3′, marker fragment length 78 bp); LSPM16 (PstI+CC: FAM-5′-GACTGCGTACATGCAG CC-3′, and MseI+CTA: 5′-GATGAGTCCTGAGTAA CTA-3′, marker fragment length 173 bp); LSPM3 (PstI+AT: FAM-5′-GACTGCGTACATGCAG AT-3′, and MseI+CTA: 5′-GATGAGTCCTGAGTAA CTA-3′, marker fragment length 113 bp estimated by electrophoresis); LSPM13 (PstI+CT: FAM-5′-GACTGCGTACATGCAG CT-3′, and MseI+CAA: 5′-GATGAGTCCTGAGTAA CAA-3′, marker fragment length 449 bp estimated by electrophoresis); LSPM7 (PstI+TA: FAM-5′-GACTGCGTACATGCAG TA-3′, and MseI+CTG: 5′-GATGAGTCCTGAGTAA CTG-3′, marker fragment length 83 bp estimated by electrophoresis); LSPM9 (PstI+TG: FAM-5′-GACTGCGTACATGCAG TG-3′, and MseI+CCT: 5′-GATGAGTCCTGAGTAA CCT-3′, marker fragment length 171 bp estimated by electrophoresis).

### BAC library construction and high density filter production

Sperm samples were freshly prepared from the dextral *L. stagnalis* and washed in phosphate-buffered saline (PBS: 0.8% NaCl, 0.02% KCl, 0.144% Na_2_HPO_4_, 0.024% KH_2_PO_4_). Then the sperm suspension was mixed with an equal volume of 1% InCert agarose (FMC Bioproducts, USA) in PBS and poured into a disposable plug mold (Bio-Rad, USA) as described previously^47^. After proteinase K digestion, a restriction digestion was performed with *Hind*III endonuclease (New England Biolabs, UK). Partially-digested DNA was put into the centre wells of a 1% agarose gel and separated by pulse field gel electrophoresis (PFGE)(CHEF-DRII; Bio-Rad) (6  V/cm, 50  s switch time, 18 hours run time, 12.5 °C). The region of the compression zone containing DNA fragments in the size range from 150 to 450 kb was excised from the unstained gel, embedded into a second gel and compressed by PFGE. The concentrated high molecular weight (>150 kb) DNA band was excised and recovered from the agarose gel slices. These DNA fragments were ligated into the pBAC-lac vector and the ligation products were used to transform the DH10B strain^48^. The resulting library provided an approximately 22-fold coverage of the *L. stagnalis* diploid genome. The entire library was gridded onto 22.5 × 22.5 cm^2^ Hybond-N+ nylon membranes (Amersham Pharmacia Biotech, USA) using the Genetix Q-Pix (Molecular Devices, USA). After transfer, membranes were baked for 2 h at 80 °C.

### Chromosome walking

The AFLP marker HTLM1 was converted into a sequence-tagged site (STS) marker for BAC screening. Chromosome walking was initiated with the 106 bp HTLM1 probe amplified from total genomic DNA using two primers, HTLM1-forward 5′-GTTCAGACTTATAACCATGATATATTG-3′, and reverse 5′-AGATCTATTGGACGACTGTAGGTTCAT-3′. The PCR probes were labelled with α^32^P-dCTP (3000 Ci/mmol) using a Ready-To-Go DNA labelling kit (Amersham Pharmacia Biotech), and the unincorporated nucleotides were removed by passage through a Sephadex G-50 column (Amersham Pharmacia Biotech). The high-density hybridization filters were prehybridized for 2 h at 68 °C in PerfectHyb hybridization solution (Toyobo, Japan), and then hybridized separately with each α^32^P-dCTP-labelled probe at 65 °C for 12 h. The blots were subsequently washed under stringent conditions (final wash: 0.1×SSC, 0.1% SDS for 30 min at 65 °C). Membranes were exposed to imaging plates (IP) (Fuji Film, Japan) or X-ray film (Kodak X-Omat, Japan) in a cassette overnight. The results were analyzed using a BAS-1800 Photo Image Scanner (Fuji Film).

Four microliters of BAC culture in LB freezing media was inoculated into 4 ml of LB media containing 12.5 μg/ml chloramphenicol and incubated for 20 h at 37 °C. BAC DNA was isolated using the Qiagen plasmid midi kit (Qiagen). The specificity of BAC clones selected by hybridization was verified by PCR with HTLM1 primers to exclude false-positive hybridizing clones.

Plasmid DNA (1 μg) was used as the template for BAC end sequencing reactions in a total volume of 20 μl. The plasmid DNAs were reacted with BigDye terminator v3.1 (Applied Biosystems, USA) using M13 universal primer (M13-forward GTAAAACGACGGCCAGT or M13-reverse GGAAACAGCTATGACCATG) for DNA sequencing of both ends of BAC inserts. DNA sequencing of these products was carried out using an ABI3130xl Genetic Analyzer (Applied Biosystems). Sequencing quality was assessed using Sequencing Analysis v5.2 software (Applied Biosystems). For BAC insert size determination, about 2 μg of the plasmid DNA was subjected to *Not*I enzyme digestion at 37 °C for 3 h and loaded onto a PFGE gel alongside the MidRange Marker II (New England Biolabs) as a size standard. On the basis of insert size and overlap length, one clone was selected for further chromosome walking. BAC clones with the largest inserts were preferentially selected to accelerate walking.

BAC screening was conducted in both directions until an overlapped clone was identified and gaps between two recombination points were filled. The basic steps employed during chromosome walking to identify BAC clones extending from the anchored clones are outlined in Fig. 1. The primers used for BAC end-probes and recombination analysis were as follows: 47M13F-forward 5′-CAAACAACATCATCGCAACA-3′, and reverse 5′-GTCCTCCTGGCAAGGTAATG-3′; 125M13F-forward 5′-TAGATGTCGCCATTTCAACG-3′, and reverse 5′-CCAATGATGAGGATGGACTTG-3′; 170M13R-forward 5′-CGTTCTGGAGACACCCTTTC-3′, and reverse 5′-TGACGGGAGTGGCAGATATT-3′; 177M13R-forward 5′-AGAATTGCATTGCCTCTTGG-3′, and reverse 5′-TCTCTCTGTTTCCACCTCACAA-3′; 177M13F-forward 5′-AATGCATTGACTTCGGTCGT-3′, and reverse 5′-GCAGACATAGCACACCCACA-3′; 204M13R-forward 5′-ATGGACAGAATAGGTGCCAGGA-3′, and reverse 5′-CCGAGTCTCCCATGGGTCAAG-3′; 232M13F-forward 5′-CCAGCTTCCGAGCCGTTATC-3′, and reverse 5′-GCTCAAAACCCTCAACCAAG-3′; 260M13F-forward 5′-GCATCGATTCTGTGTGTTGG-3′, and reverse 5′-AAAGTGAGTACCGGCATTGG-3′; 278M13R-forward 5′-CATTTGCCTATTTTGGCTCAG-3′, and reverse 5′-AATGCTTGAGGCTGTTCGTT-3′; 303M13F-forward 5′-AGGGTATTTCAAATCGAGCA-3′, and reverse 5′-GCGGTTGCAACTGTCTTTGT-3′.

### DNA sequence of the BAC clones

Screened BAC clone DNA was purified by CsCl gradient ultracentrifugation and sheared to 4 kb DNA fragments using a GeneMachine HydroShear (GeneMachine, USA). The DNA fragments were treated with Klenow fragment, T4 DNA polymerase, and polynucleotide kinase and then were cloned into the pUC19 plasmid vector. The ligation products were used to transform *Escherichia coli* strain DH5a. We picked 500-1000 *E. coli* transformants for each BAC DNA, isolated and sequenced the DNA. Trace data obtained by using the Applied Biosystems 3130xl Genetic Analyzer were assembled with PhredPhrapConsed. The DNA sequence of overlap regions between adjacent BAC clones was determined with shotgun clones derived from both BAC clones. In addition, gap filling was performed using direct sequencing of BAC clones and long range PCR. Furthermore, the sequence of the non-recombination region covered by the seven BAC DNA clones was analyzed on the Illumina HiSeq 2000. The non-recombination region restricted between the two fine mapping markers, BAC170fm1 and BAC303fm2, was separated by a small gap. This DNA region was only about 100 bp long, but it resisted sequencing by any method. Finally, we identified the non-recombination genome region to be about 780 kb in length.

### Fine mapping

The non-recombination region in the genetic map specified by a series of BAC DNA clones isolated by chromosome walking, allowed the genetic map to be converted into a physical map by sequencing of these clones. Thus, it became possible to identify the recombination breakpoints in more detail by sequence-based analysis. On the HTLM1 side, hot spots of meiotic recombination were in the region between BAC end marker 125M13F and 170M13R, which were found by the process of chromosome walking. Sequence-based markers 170 fm1 and 170 fm2 for the analysis of SNP patterns between the dextral (DD) and sinistral (dd) homozygotes were added in the area (Fig. 1a), and recombination breakpoints were found between 170 fm1 and 177M13R in seven of the recombinant individuals (Supplementary Table S3). On the HTLM2 side, recombination points had been found between the BAC end marker 278M13R and 303M13F, then fine mapping markers 303 fm1 and 303 fm2 were added in the area and SNP patterns were analyzed (Fig. 1a). Recombination breakpoints were identified between 303 fm1 and 303 fm2 in two of the recombinant individuals (Supplementary Table S3). The primers used for fine mapping were as follows: 170 fm1-forward 5′-TGGTTTCCATGCACAAAGTG-3′, and reverse 5′-TCCATTGTCATTGCCTTCAA-3′ (225 bp); 170 fm2-forward 5′-TGCAGGGGAAGATTTGATATG-3′, and reverse 5′-GCAGTTACAGGCCTTGCCTA-3′ (430 bp); 303 fm1-forward 5′-CCCTCTGACGTGCATCTGTA-3′, and reverse 5′-CCCTTCCTACGCCACTGTTA-3′ (248 bp); 303 fm2-forward 5′-GCATATGTGTTGGTGCAAGG-3′, and reverse 5′-CGAGTACCCAGGTCTGCATT-3′ (405 bp).

### Ab initio gene prediction

In the annotation process, RepeatMasker (http://www.repeatmasker.org/) was used to mask the repetitive regions^49^. Gene prediction was carried out using GENSCAN (http://genes.mit.edu/GENSCAN.html)^24^. Furthermore, a BLAST search was carried out against a non-redundant data set available at NCBI. All predicted transcription units were compared to both genomic and transcriptomic sequences from *Biomphalaria glablata* and *Aplysia california* to identify the homologue genes. Any candidate with a significant match to retrotransposon simple repeats was removed according to the gene definition. Exon regions were identified using GeneWise (http://www.ebi.ac.uk/Tools/psa/genewise/) and confirmed using FGENESH (http://www.softberry.com/berry.phtml?topic=fgenesh&group=programs&subgroup=gfind)^50^ was supportively used to confirm this data.

### Representative candidate genes for snail handedness determination

On the basis of the positional candidate approach, 15 candidate genes were mapped within the nonrecombinant interval (780 kbp) (Fig. 1b). Quantitative RT-PCR analysis of all candidate genes narrowed the candidates to 13 maternally expressed genes. We have previously accumulated evidence that the spiral deformation and spindle inclination are uniquely observed only in the dominant dextral embryos, and they strongly depend on actin cytoskeletal dynamics^21^,^22^. Therefore, we assumed that an actin regulatory gene is the most logical candidate for snail chirality determination. On this basis, out of 13 maternally expressed genes, five genes, namely *dia1, dia2, mycbp2, fat*, and *fry*, were selected as the strong candidates although at this stage we already knew that *Lsdia1* was hardly expressed in recessive sinistral strains. The cDNA sequences of all five genes expressed in 1-cell embryos maternally were determined and compared between the dextral and sinistral strains. The full-length sequences were obtained by 5′/3′-RACE using the SMARTer RACE cDNA Amplification Kit (TaKaRa) according to the manufacturer’s protocol. The biological profiles of these genes and observations reported here for the *Lymnaea stagnalis* (Ls) genes are summarized below.

#### Lsdia1

Here we showed that *Lsdia1* is hardly expressed in recessive sinistral embryos compared with dominant dextral ones. This was confirmed at both mRNA and protein levels. c.184delC was detected only in sinistral strains. This mutation causes a frameshift after amino acid 62 and premature termination after amino acid 85 (p.Leu62Serfs*24). The GenBank accession numbers for the both *Lsdia1* sequences are KX387869 and KX387870.

#### Lsdia2

By quantitative expression analysis using quantitative RT-PCR and Northern blot we showed no differences in *Lsdia2* mRNA expression between dextral and sinistral embryos. In addition, there were no differences between the *Lsdia2* sequences of dextral and sinistral strains. *Lsdia2* is located in the boundary position between recombinant and nonrecombinant region, and a large part of *Lsdia2* is excluded from the target locus for handedness determination. The GenBank accession numbers for the both *Lsdia2* sequences are KX387871 and KX387872.

#### Lsmycbp2

Quantitative RT-PCR analysis here showed no differences in *Lsmycbp2* mRNA expression between dextral and sinistral strains. Two amino acid differences were detected at the protein level, but these amino acids were not evolutionary conserved. The GenBank accession numbers for the both *Lsmycbp2* sequences are KX387877 and KX387878.

#### Lsfat

Quantitative RT-PCR analysis showed no differences in *fat* mRNA expression between dextral and sinistral strains. *Lsfat* is located in the boundary position between the recombinant and non-recombinant region, and a large part of *fat* is excluded from the target locus for snail handedness determination. Out of 19 amino acid differences, 8 were detected in target locus, but these amino acids were not evolutionary conserved. The GenBank accession numbers for the both *Lsfat* sequences are KX387879 and KX387880.

#### Lsfry

Quantitative RT-PCR analysis showed no differences in *fry* mRNA expression between dextral and sinistral strains. One amino acid difference was detected at protein level, but this amino acid was not evolutionary conserved. The GenBank accession numbers for the both *Lsfry* sequences are KX387881 and KX387882.

### cDNA cloning of Dia homologs from other snail species and of *L.stagnalis* cytoskeletal genes

*Diaphanous* homologous genes were also identified in snail species, *L. peregra, P. acuta* and *I. exustus*. On the basis of findings in *L. stagnalis*, these genes were expected to be expressed in large amounts in the gonads. Thus, we isolated gonads from adult snails of these species, and total RNA was extracted using TRIzol reagent (Invitrogen). RT-PCR and 5′/3′-RACE were performed by using cDNA derived from the gonad tissues. First, their partial fragments of FH3 (DID + DD) and FH2 domain were amplified by using each degenerate primer set and the sequences were identified. Next, their full-length sequences were obtained by 5′/3′-RACE and amplification of the internal region between the identified partial sequences of FH3 and FH2. In *L. peregra*, two isotype genes, *Lpdia1* and *Lpdia2*, were found similarly to *L. stagnalis*. On the other hand, only one *dia* gene (more closely homologous to *Lsdia2* than *Lsdia1*) was present and could be cloned from each of *P. acuta (Padia*) and *I. exustus (Iedia*). The GenBank accession numbers for our sequences of *Lpdia1, Lpdia2, Padia* and *Iedia* are KX387873 - KX387876. The primers used for cloning of the partial sequences of Dia family genes are as follows: FH3 domain-Fw1 5′-GCGCGGAATTC ATGCARGTNGCNTGYATHCA-3′ and Rv1 5′-GCGCGCAAGCTT TCYTCYTTRTGYTCRTGRAA-3′; For *L. peregra* and *P. acuta*, FH2 domain-Fw2 5′-GCGCGGAATTC ATGGARGARTTYTTYGGNGA-3′ and Rv3 5′-GCGCGCAAGCTT TTRTCCATNACNCCYTCYTG-3′: For *I. exusts*, FH2 domain-Fw3 5′-GCGCGGAATTC GCNAARGARGCNAARGARAA-3′: Rv 3 5′-GCGCGGAATTC ATGGARGARTTYTTYGGNGA-3′.

In *L. stagnalis*, the sequences of cytoskeletal β-actin and β-tubulin genes, *Lsactb* and *Lstubb*, were identified for use as an internal control. Two isotypes were found for each gene in the both dextral and sinistral 1cell embryos by RT-PCR and 5′/3′-RACE cloning. The GenBank accession numbers for the sequences are KX387883 - KX387890. The primers used for cloning of the partial sequence of *Lsactb* and *Lstubb* are as follows: LsActb-deg-Fw 5′-GGNTTYGCNGGNGAYGAYGC-3′ and Rv 5′-ACNGCYTGDATNGCNACRTACAT-3′; LsTubb-deg-Fw 5′-AAYAAYTGGGCNAARGGNCAYTA-3′ and Rv 5′-CATYTGYTCRTCNACYTCYTTCAT-3′.

### Quantitative RT-PCR

Total RNA was isolated from the oocytes, embryos or organ tissues according to the manufacturer’s instructions with TRIzol Reagent (Invitrogen, USA). The total RNA was reverse-transcribed into cDNA using the SuperScript III First Strand cDNA synthesis system (Invitrogen). Each of the cDNA samples was used as a template for real-time PCR using SYBR Premix Ex Taq II (TaKaRa, Japan) on the ABI PRISM 7000 Sequence Detection System (Applied Biosystems). The primers for identification of the maternal expression of predicted gene fragments were constructed (at least two sets per gene) on the basis of exon-intron information predicted for the handedness-determining locus by GeneWise program (http://www.ebi.ac.uk/Tools/psa/genewise/)^51^. Each cycle consisted of initial denaturation at 95 °C for 30 s, followed by 40 cycles of 95 °C for 5 s and 60 °C for 30 s. *Lstubb1* or *Lsactb* was used as an endogenous control to normalize each sample. All reactions were performed in duplicate. The RNA expression level of diaphanous gene in dextral 1-cell embryos was adjusted as the base line (1.0-fold) and the relative expression levels in sinistral embryos and/or at other developmental stages were determined accordingly. The primers used for quantification of the *Lsdia1* and *Lsdia2* mRNA in early embryos were as follows: Lsactb-forward 5′-TCACACTGTTCCCATCTATGAAGGCT-3′ and reverse 5′-GGGCACCTGAATCTCTCATTACCAAT-3′; Lstubb1-forward 5′-GTGCTAAGTTCTGGGAGGTGATCTCA-3′ and reverse 5′-CATATTTTCCACCTGATGCCTCATT-3′; Lsdia1-forward 5′-GTTCATTCAAAATAGCCTGAACAT-3′ and reverse 5′-GCTAGATACCCAAAATTGGAAAGT-3′; Lsdia2-forward 5′-AGCAGATGATGAGTCAGAAACAG-3′ and reverse 5′-ATAACAACATATTCAGCCCTGCT-3′.

### Northern blot analysis

For Northern analyses, 20 μg of total RNA from 1-cell embryos was electrophoresed in formaldehyde agarose (0.7%, w/v) denaturing gels in 1×MOPS running buffer (20 mM MOPS, pH 7.0, 8 mM acetate and 1 mM EDTA). The RNA was then transferred on to Hybond-N+ nylon membranes (Amersham Pharmacia Biotech) by the capillary blotting method using 20×SSC (0.3 M trisodium citrate and 3.0 M sodium chloride) as the transfer buffer. After transfer, membranes were baked for 2 h at 80 °C and prehybridized for 2.5 h at 68 °C in PerfectHyb hybridization solution (Toyobo).

Three cDNA probes were prepared by reverse transcription and PCR as follows. The first strand cDNA was synthesized with using the SuperScript III First Strand cDNA synthesis system (Invitrogen) and was amplified with ExTaq DNA polymerase (TaKaRa). The three primer sets used for probe synthesis were as follows: Lsdia1-3′UTR-forward 5′-TCTTTTATAGTGAAATGAATATTGCTACG-3′and reverse 5′-AAAATATAAAGACTTTGTTGGGCTCAT-3′; Lsdia2-3′UTR-forward 5′-AAGATGGCAAGGGATTCAAAGTGTT-3′ and reverse 5′-TTCATGACCAAAGTGGGAGGAATTA-3′; Lsdia1-CDS-forward 5′-AGAGTTTACCTCTTGATGACCA-3′ and reverse 5′-CAGGGTCCATCAAACTATTGATAA-3′.

Amplified probes were labelled with α^32^P-dCTP (3000 Ci/mmol) using the Ready-To-Go DNA labelling kit (Amersham Pharmacia Biotech) and the unincorporated nucleotides were removed by passage through a Sephadex G-50 column (Amersham Pharmacia Biotech). The membranes were hybridized separately with each α^32^P-dCTP labelled probe in the same buffer at 65 °C for 16 h. Blots were subsequently washed under stringent conditions (final wash: 0.2×SSC, 0.2% SDS for 10 min at 65 °C). Membranes were exposed to IP (Fuji Film) in a cassette overnight. The results were analyzed using a BAS-1800 Photo Image Scanner (Fuji Film).

### Antibodies

To examine Dia protein expression, we sought to make antibodies that specifically recognize LsDia1 and discriminate it from LsDia2. Given that the amino acid sequences of both proteins are very similar, we focused on differences in the amino acid sequence of the C-terminal region. Anti-LsDia1 antibodies (ST0301 and ST0302) were produced by immunizing rabbits with the synthetic peptide SSVDQEKLKHKKKKH corresponding the C-terminal region of LsDia1 (amino acids 1054–1068), and purified by affinity column conjugated with the synthetic peptide KLKHKKKKH (amino acids 1060–1068) (Fig. 3b). Both polyclonal antibodies were suitable for the detection of LsDia1 protein by Western blotting. The polyclonal antibodies were produced by Sigma Aldrich Inc. in accordance with relevant guidelines and regulations.

### SDS-PAGE and Western blotting

Embryos at each early developmental stage were collected by taking them out of the capsules in 5×HF solution, and transferring them to plastic tubes. After removing as much liquid as possible, the tubes were frozen immediately in liquid nitrogen and stored at −80 °C. Each stage samples for SDS-PAGE were prepared on ice and contained 50 embryos in 10 μl of SDS PAGE sample buffer [125 mM Tris-HCl, pH 6.8, 4% SDS, 20% glycerol, 2% 2-mercaptoethanol, 1×Protease Inhibitor Cocktail (Roche)] with a small amount of bromophenol blue. The SDS-PAGE samples were heated for 3 minutes at 98 °C to extract proteins from the embryos and denature them. The total volume of each sample was loaded and run on a 9.0% SDS-polyacrylamide gel. The separated proteins were transferred to a Hybond P PVDF membrane (GE Healthcare) by semi-dry electrotransfer. The membranes were blocked with TBST (50 mM Tris-HCl, pH 7.5, 150 mM NaCl 0.1% Tween20) containing ECL Advance Blocking Reagent (GE Healthcare) at room temperature for 1 hour and then incubated with the primary antibody anti-LsDial diluted by 1:10000 in blocking buffer at 4 °C overnight. The membranes were washed and incubated with Horseradish Peroxidase conjugated anti-rabbit antibody (GE Healthcare) diluted by 1:50000 in blocking buffer at room temperature for 1 hour. Bands on washed membranes were detected by ECL Advance Western Blotting Detection substrate (GE healthcare). Subsequently, after the membranes were treated with stripping buffer (0.2 M Glycine-HCl, pH 2.8), β-Tubulin was detected as internal control by similar procedure using the monoclonal anti-β-tubulin antibody (TUB2.1, Sigma) diluted by 1:20000 and Horseradish Peroxidase conjugated anti-mouse antibody (GE Healthcare) diluted by 1:50000 in blocking buffer. The chemiluminescence signals were imaged using ChemiDoc MP Imaging System (Bio-Rad), and the images were analyzed using Image Lab Software (Bio-Rad).

### Whole mount *in situ* hybridization

High selectivity *in situ* hybridization experiments of *L. stagnalis* embryos were performed as we described previously^13^. Briefly, the protocol was constructed by incorporating a method to remove most of the non-specific hybridization. This protocol can be applied to analyses of the expression patterns of various genes through developmental stages with only slight customization depending on the type of probe (e.g., probe length, probe concentration, hybridization temperature, washing condition).

Egg mass immediately after oviposition was collected from aquarium maintaining adult snails. The egg capsules were isolated from surrounding jelly mass by rolling them on a sheet of filter paper^44^, and cultured in 1.5×HF solution at 25 °C until they reached to the respective developmental stages according to the timetable described by Biggelaar^52^. Each of the early cleaving embryos were carefully taken out from their egg capsule using two pairs of sharp tweezers in 5×HF solution. The collected embryos were transferred to new 5×HF solution by a glass capillary and gently washed to remove adhering albumin. Embryos were fixed with 4.0% paraformaldehyde in MTSTr (10 mM PIPES-KOH, pH 6.9, 5 mM EGTA, 30 mM KCl, 5 mM MgCl_2_, 0.1% Triton X-100) at 4 °C overnight. The fixed embryos were washed in PBSTw (1×PBS, pH 7.0, 0.1% Tween 20), processed by sequential treatment with MeOH in PBS (50%, 100%) and stored in 100% MeOH at −30 °C.

For *in situ* hybridization, embryos were rehydrated gradually through a series of MeOH in PBS (75%, 50%, 25%) and embryos at each stage from 1 to 24-cell were collected with PBSTw into 4-well dishes for each type of probe. WISH experiments were performed using 4-well dishes thereafter. The protocols up to prehybridization followed the method reported by Nederbragt^53^ except for the Hybridization buffer (HB) [5×SSC, pH 7.0, 50% formamide, 5 mM EDTA, 100 μg/ml yeast tRNA, 100 μg/ml heparin, 1×Denhardt’s solution (Wako), 0.1% Tween 20, 0.1% CHAPS].

Digoxigenin (Dig)-labeled RNA probes were synthesized by *in vitro* transcription with Dig RNA labeling mix (Roche) from PCR-amplified templates adding the T7 RNA polymerase recognition sequence at one end. The primers used for amplification from a recombinant plasmid DNA were as follows: LsDia1-N antisense probe (1002 nt)-Fw: 5′-ATGTTTTTCAACAAAAAAGGCAGAA-3′ and Rv: 5′-TAATACGACTCACTATAGGG CTCCATGGTTATGTTGTCATAGCGA-3′;

LsDia1-N sense probe (1002 nt)-Fw: 5′-TAATACGACTCACTATAGGGATGTTTTTCAACAAAAAAGGCAGAA-3′ and 5′-CTCCATGGTTATGTTGTCATAGCGA-3′; LsDia2-N antisense probe (1002 nt)-Fw: 5′-ATGTTTTTCAACAAGAAAAGCAAAA-3′ and Rv: 5′-TAATACGACTCACTATAGGG CTCCATGCTTATGTTGTCATAGCGA-3′; LsDia2-N sense probe (1002 nt)-Fw: 5′-TAATACGACTCACTATAGGG ATGTTTTTCAACAAGAAAAGCAAAA-3′ and Rv: 5′-CTCCATGCTTATGTTGTCATAGCGA-3′; LsActb1 WISH antisense probe (525 nt)-Fw: 5′-CACGGTATCGTCACCAACTGGGATG-3′ and Rv: 5′-TAATACGACTCACTATAGGG GACTTGACCGTCGGGAAGCTCGTAG-3′; LsActb1 WISH sense probe (525 nt)-Fw: 5′-TAATACGACTCACTATAGGG CACGGTATCGTCACCAACTGGGATG-3′ and Rv: 5′-GACTTGACCGTCGGGAAGCTCGTAG-3′; LsTubb1 WISH antisense probe (479 nt)-Fw: 5′-AACAACTGGGCCAAAGGTCACTACA-3′ and Rv: 5′-TAATACGACTCACTATAGGG ACCATGTTGACAGCCAGTTTTCTCA-3′; LsTubb1 WISH sense probe (479 nt)-Fw: 5′-TAATACGACTCACTATAGGG AACAACTGGGCCAAAGGTCACTACA-3′ and Rv: 5′-ACCATGTTGACAGCCAGTTTTCTCA-3′

Hybridization was performed in HB with 1.0 ng/μl denatured probe at 55 °C for 24 h. Embryos were washed in fresh HB twice for 1 h 2 times at 55 °C, HB/2×SSCTw (2×SSC, pH 7.0, 0.1% Tween 20) (50:50) for 10 min at 55 °C, and then 2×SSCTw for 5 min 3 times at RT. Subsequently, to remove non-specifically bound probe, embryos were treated with 20 μg/ml RNaseA for 30 min at 37 °C, and then washed twice each time for 30 min with Hybridization wash buffer (2×SSC, pH 7.0, 50% formamide, 0.1% Tween 20), twice each time for 10 min with SSCTw, and finally twice each time for 30 min with 0.2×SSC containing 0.3% Triton X-100 at 55 °C.

Embryos were blocked with blocking solution (Roche) at RT for 1 h and then incubated with the Anti-DIG-AP, Fab fragments (Roche) diluted by 1:1000 in blocking solution at 4 °C overnight. After washing, the embryos were stained in BM purple solution (Roche) at RT or 30 °C for several hours with similar conditions for the antisense and sense probe of same target gene. The stained embryos were washed in PBSTw and refixed by 4% paraformaldehyde-MTSTr to stop alkaline phosphatase activity. After washing through a series of MeOH solutions, the embryos were stained with DAPI in PBSTw and cleared in 80% glycerol. Stained embryo images were acquired on a Zeiss Axioskop2 microscope equipped with a Zeiss Axiocam MRc CCD camera.

### Immunostaining

Immunostaining was performed as described previously^21^. Images were obtained using a Zeiss LSM5 pascal conforcal microscope equipped with a 20×objective lens and processed with Zeiss LSM Image Browser software.

## Additional Information

**How to cite this article**: Kuroda, R. *et al. Diaphanous* gene mutation affects spiral cleavage and chirality in snails. *Sci. Rep.* **6**, 34809; doi: 10.1038/srep34809 (2016).

## Supplementary Material

Supplementary Information
