# Supplementary material for: Diaphanous gene mutation affects spiral cleavage and chirality in snails
Source: Sci Rep. 2016 Oct 6;6:34809. doi: 10.1038/srep34809 (PMC5052593; doi:10.1038/srep34809)
Supplement: Supplementary Information [file srep34809-s1.doc]

**Supplementary data**

***Diaphanous* gene mutation affects spiral cleavage and chirality in snails**

Reiko Kuroda^1,2,3,4,5^*, Kohei Fujikura^1,3^, Masanori Abe^2,3,4^, Yuji Hosoiri^1,3^, Shuichi Asakawa^6^, Miho Shimizu^3^, Shin Umeda^5^, Futaba Ichikawa^5^, & Hiromi Takahashi^5^.

^1^ Department of Life Sciences, Graduate School of Arts and Sciences, The University of Tokyo

^2^ Department of Biophysics and Biochemistry, Graduate School of Science, The University of Tokyo

^3^ JST ERATO-SORST Kuroda Chiromorphology Project

^4^ Research Institute for Science and Technology, Tokyo University of Science

^5^ Department of Applied Biological Science, Graduate School of Science and Technology, Tokyo University of Science

^6^ Department of Molecular Biology, Keio University School of Medicine

*Corresponding Author:

Reiko Kuroda; Research Institute for Science and Technology, Tokyo University of Science, 2641 Yamazaki, Noda-shi, Chiba 278-8510 Japan. e-mail: rkuroda@rs.tus.ac.jp

**Materials**

***Snails***

Adult freshwater snails of *L. stagnalis* and *peregra* have been reared in our laboratory over many years, essentially as described earlier^1^. They are maintained at 20-22°C and given fresh lettuce leaves and artificial food for tropical fish. The snails can produce one clutch of eggs almost every day under our laboratory conditions. *Indoplanobis exustus* was kindly supplied by Meitousuien Remix Co. Ltd. (Nagoya, Japan) and *Physa acuta* was collected at a pond in Risoukai Nature Park of Tokyo University of Science. They were maintained as above for other pond snails.

**Biological profiles of candidate genes**

On the basis of the positional candidate approach, 15 candidate genes were mapped within the nonrecombinant interval (780 kbp) (Fig. 1b). Quantitative RT-PCR analysis of all candidate genes narrowed the candidates to 13 maternally expressed genes. We have previously accumulated evidence that the spiral deformation and spindle inclination are uniquely observed only in the dominant dextral embryos, and they strongly depend on actin cytoskeletal dynamics^2,3^. Therefore, we assumed that an actin regulatory gene is the most logical candidate for snail chirality determination. On this basis, out of 13 maternally expressed genes, five genes, namely *dia1*, *dia2*, *mycbp2*, *fat*, and *fry*, were selected as the strong candidates although at this stage we already knew that *Lsdia1* was hardly expressed in recessive sinistral strains. The biological profiles of these genes are summarized below.

*dia1* and *dia2*

The diaphanous-related formin proteins constitute a subfamily of Rho GTPase-binding formin homology proteins conserved between vertebrates and invertebrates^4-10^. The diaphanous-related formins nucleate and assemble unbranched actin structures^7-14^, therefore they are implicated in various actin-based cellular functions, including cytokinesis, cell polarity, cell morphogenesis and cell migration^15-17^. The diaphanous-related formins also regulates microtubule (MT) stabilization and the crosstalk between the actin and MT cytoskeletons^18,19^. In humans, mDia family proteins are reported to localize to the spindle microtubules during mitosis^20^, cause reorientation of the microtubule-organizing center (MTOC)^21,22^, and associate with the kinetochore to contribute to chromosome alignment^23^. Therefore, the actin-related diaphanous gene(s) *Lsdia* was assumed to be the strongest candidate for the handedness-determining gene.

*mycbp2*

Mycbp2 (MYC Binding Protein 2), also known as PAM (Protein Associated with Myc), and its orthologs are conserved from fly to mammals^24^, and function as E3 ubiquitin-protein ligases for regulating development of the nervous system across species^24-26^. Mycbp2 has been reported to interact directly with F-actin *in vitro*^27^, and to behave as a GEF (guanine nucleotide exchange factor) for small GTPase Ran which is known to be involved in the formation of the mitotic spindle^28^. Thus, Mycbp2 has the potential to be involved in the regulation of the spiral deformation and spindle inclination.

*Fat*

Fat is an atypical cadherin, which is characterized by its large extracellular domain containing 34 cadherin repeats^29,30^. Fat was reported as a tumor suppressor gene essential for regulating cell proliferation during *Drosophila* development^31,32^. Recently, it has been revealed that its function is related to regulation of planar cell polarity (PCP) by regulating actin dynamics and cell-cell contacts at the cell surface^30,33-36^. Therefore, fat is also a candidate gene.

*Fry*

Furry (Fry) protein is evolutionarily conserved in eukaryotic species including yeast, nematodes, fruit fly and mammals broadly^37^. It has been revealed that Fry and its orthologues interact with nuclear Dbf2-related (NDR) kinases and Fry-NDR signaling regulates cellular morphogenesis, neuronal development and cell division^37-41^. Although, direct interaction between Fry and actin cytoskeleton has not been reported so far, recent studies suggest that Fry orthologues appear to form a complex with F-actin during cell morphogenesis in yeast^42,43^. Fry and NDR kinase are also involved in regulating the mitotic spindle organization and chromosome alignment in human cell^44^.

**Reference**

1. Kuroda, R., Endo, B., Abe, M. & Shimizu, M. Chiral blastomere arrangement dictates zygotic left-right asymmetry pathway in snails. *Nature* **462**, 790-794 (2009).

2. Kuroda R. How a single gene twists a snail. *Integr. Comp. Biol.* **54**, 677-687 (2014).

3. Shibazaki, Y., Shimizu, M. & Kuroda, R. Body handedness is directed by genetically determined cytoskeletal dynamics in the early embryo. *Curr. Biol*. **14**, 1462-1467 (2004).

4. Castrillon, D. H. & Wasserman, S. A. *Diaphanous* is required for cytokinesis in Drosophila and shares domains of similarity with the products of the limb deformity gene. *Development* **120**, 3367-3377 (1994).

5. Rivero, F. *et al*. A comparative sequence analysis reveals a common GBD/FH3-FH1-FH2-DAD architecture in formins from Dictyostelium, fungi and metazoa. *BMC Genomics* **6**, 28 (2005).

6. Chalkia, D., Nikolaidis, N., Makalowski, W., Klein, J. & Nei, M. Origins and evolution of the formin multigene family that is involved in the formation of actin filaments. *Mol. Biol. Evol.* **25**, 2717-2733 (2008).

7. Schönichen, A. & Geyer, M. Fifteen formins for an actin filament: a molecular view on the regulation of human formins. *Biochim. Biophys. Acta* 1803, 152-163 (2010).

8. Aspenström, P. Formin-binding proteins: modulators of formin-dependent actin polymerization. *Biochim. Biophys. Acta* **1803,** 174-182 (2010).

9. Chesarone, M. A., DuPage, A. G. & Goode, B. L. Unleashing formins to remodel the actin and microtubule cytoskeletons. *Nat. Rev. Mol. Cell Biol*. 11, 62-74 (2010).

10. Breitsprecher, D. & Goode, B. L. Formins at a glance. *J. Cell Sci*. **126**, 1-7 (2013).

11. Watanabe, N., Kato, T., Fujita, A., Ishizaki, T. & Narumiya, S. Cooperation between mDia1 and ROCK in Rho-induced actin reorganization. *Nat. Cell Biol.* 1, 136-143 (1999).

12. Tominaga, T. *et al*. Diaphanous-related formins bridge Rho GTPase and Src tyrosine kinase signaling. *Mol. Cell* **5**, 13-25 (2000).

13. Li, F. & Higgs, H. N. The mouse Formin mDia1 is a potent actin nucleation factor regulated by autoinhibition. *Curr. Biol.* **5**, 1335-1340 (2003).

14. Higashida, C. *et al*. Actin polymerization-driven molecular movement of mDia1 in living cells. *Science* **303**, 2007-2010 (2004).

15. Chhabra, E. S. & Higgs, H. N. The many faces of actin: matching assembly factors with cellular structures. *Nat. Cell Biol*. **9**, 1110-1121 (2007).

16. Shi, Y. *et al*. The mDial formin is required for neutrophil polarization, migration, and activation of the LARG/RhoA/ROCK signaling axis during chemotaxis. *J. Immunol.* **182**, 3837-3845 (2009).

17. Bogdan, S., Schultz, J. & Grosshans, J. Formin' cellular structures: Physiological roles of Diaphanous (Dia) in actin dynamics. *Commun. Integr. Biol*. **6,** e27634 (2013).

18. Ishizaki, T. *et al*. Coordination of microtubules and the actin cytoskeleton by the Rho effector mDia1. *Nat. Cell Biol*. **3**, 8-14 (2001).

19. Bartolini, F. & Gundersen, G. G. Formins and microtubules. *Biochim. Biophys. Acta* **1803**, 164-173 (2010).

20. Kato, T. *et al*. Localization of a mammalian homolog of diaphanous, mDia1, to the mitotic spindle in HeLa cells. *J. Cell Sci*. **114**, 775-784 (2001).

21. Palazzo, A. F. *et al*. Cdc42, dynein, and dynactin regulate MTOC reorientation independent of Rho-regulated microtubule stabilization. *Curr. Biol.* **11**, 1536-1541 (2001).

22. Dong, B. *et al*. Mammalian diaphanous-related formin 1 regulates GSK3β-dependent microtubule dynamics required for T cell migratory polarization. *PLoS One*. **8**, e80500 (2013).

23. Yasuda, S. Cdc42 and mDia3 regulate microtubule attachment to kinetochores. *Nature* **428**, 767-771 (2004).

24. Grill, B. Murphey, R. K. & Borgen, M. A. The PHR proteins: intracellular signaling hubs in neuronal development and axon degeneration. *Neural Dev.* **11**, 8 (2016).

25. Guo, Q., Xie, J., Dang, C. V., Liu, E. T. & Bishop, J. M. Identification of a large Myc-binding protein that contains RCC1-like repeats. *Proc. Natl. Acad. Sci.* **95**, 9172-9177 (1998).

26. Murthy, V. *et al*. Pam and its ortholog highwire interact with and may negatively regulate the TSC1.TSC2 complex. *J. Biol. Chem*. **279**, 1351-1358 (2004).

27. Pierre, S. *et al*. Toponomics analysis of functional interactions of the ubiquitin ligase PAM (Protein Associated with Myc) during spinal nociceptive processing. *Mol*. *Cell Proteomics*. **7**, 2475-2485 (2008).

28. Dörr, A. *et al*. MYCBP2 is a guanosine exchange factor for Ran protein and determines its localization in neurons of dorsal root ganglia. *J. Biol. Chem*. **290**, 25620-25635 (2015).

29. Nollet, F., Kools, P. & van Roy, F. Phylogenetic analysis of the cadherin superfamily allows identification of six major subfamilies besides several solitary members. *J. Mol. Biol*. **299**, 551-572 (2000).

30. Tanoue, T. & Takeichi, M. New insights into Fat cadherins. *J. Cell Sci*. **118**, 2347-2353 (2005).

31. Mahoney, P. A. *et al*. The fat tumor suppressor gene in Drosophila encodes a novel member of the cadherin gene superfamily. *Cell* **67**, 853-868 (1991).

32. Silva, E., Tsatskis, Y., Gardano, L., Tapon, N. & McNeill, H. The tumor-suppressor gene fat controls tissue growth upstream of expanded in the hippo signaling pathway. *Curr. Biol*. **16**, 2081-2089 (2006).

33. Moeller, M. J. *et al*. Protocadherin FAT1 binds Ena/VASP proteins and is necessary for actin dynamics and cell polarization. *EMBO J*. **23**, 3769-3779 (2004).

34. Tanoue, T. & Takeichi, M. Mammalian Fat1 cadherin regulates actin dynamics and cell-cell contact. *J. Cell Biol.* **165**, 517-528 (2004).

35. Thomas, C. & Strutt, D. The roles of the cadherins Fat and Dachsous in planar polarity specification in Drosophila. *Dev. Dyn*. **241**, 27-39 (2012).

36. Matis, M. & Axelrod, J. D. Regulation of PCP by the Fat signaling pathway. *Genes Dev*. **27**, 2207-2220 (2013).

37. Nagai, T. & Mizuno, K. Multifaceted roles of Furry proteins in invertebrates and vertebrates. *J. Biochem.* **155**, 137-46 (2014).

38. Cong, J. *et al*. The furry gene of Drosophila is important for maintaining the integrity of cellular extensions during morphogenesis. *Development* **128**, 2793-802 (2001).

39. Emoto, K. *et al*. Control of dendritic branching and tiling by the Tricornered-kinase/Furry signaling pathway in Drosophila sensory neurons. *Cell* **119**, 245-256 (2004).

40. Hergovich, A, Stegert, M. R., Schmitz, D. & Hemmings, B. A. NDR kinases regulate essential cell processes from yeast to humans. *Nat. Rev. Mol. Cell Biol.* **7**, 253-264 (2006).

41. Emoto, K. The growing role of the Hippo-NDR kinase signalling in neuronal development and disease. *J. Biochem.* **150**, 133-141 (2011).

42. Du, L. L. & Novick, P. Pag1p, a novel protein associated with protein kinase Cbk1p, is required for cell morphogenesis and proliferation in Saccharomyces cerevisiae. *Mol. Biol. Cell* **13**, 503-514 (2002).

43. Hirata, D. *et al*. Fission yeast Mor2/Cps12, a protein similar to Drosophila Furry, is essential for cell morphogenesis and its mutation induces Wee1-dependent G(2) delay. *EMBO J*. **21**, 4863-4874 (2002).

44. Chiba, S., Ikeda, M., Katsunuma, K., Ohashi, K., and Mizuno, K. MST2-and Furry-mediated activation of NDR1 kinase is critical for precise alignment of mitotic chromosomes. *Curr. Biol*. **19**, 675-681 (2009).


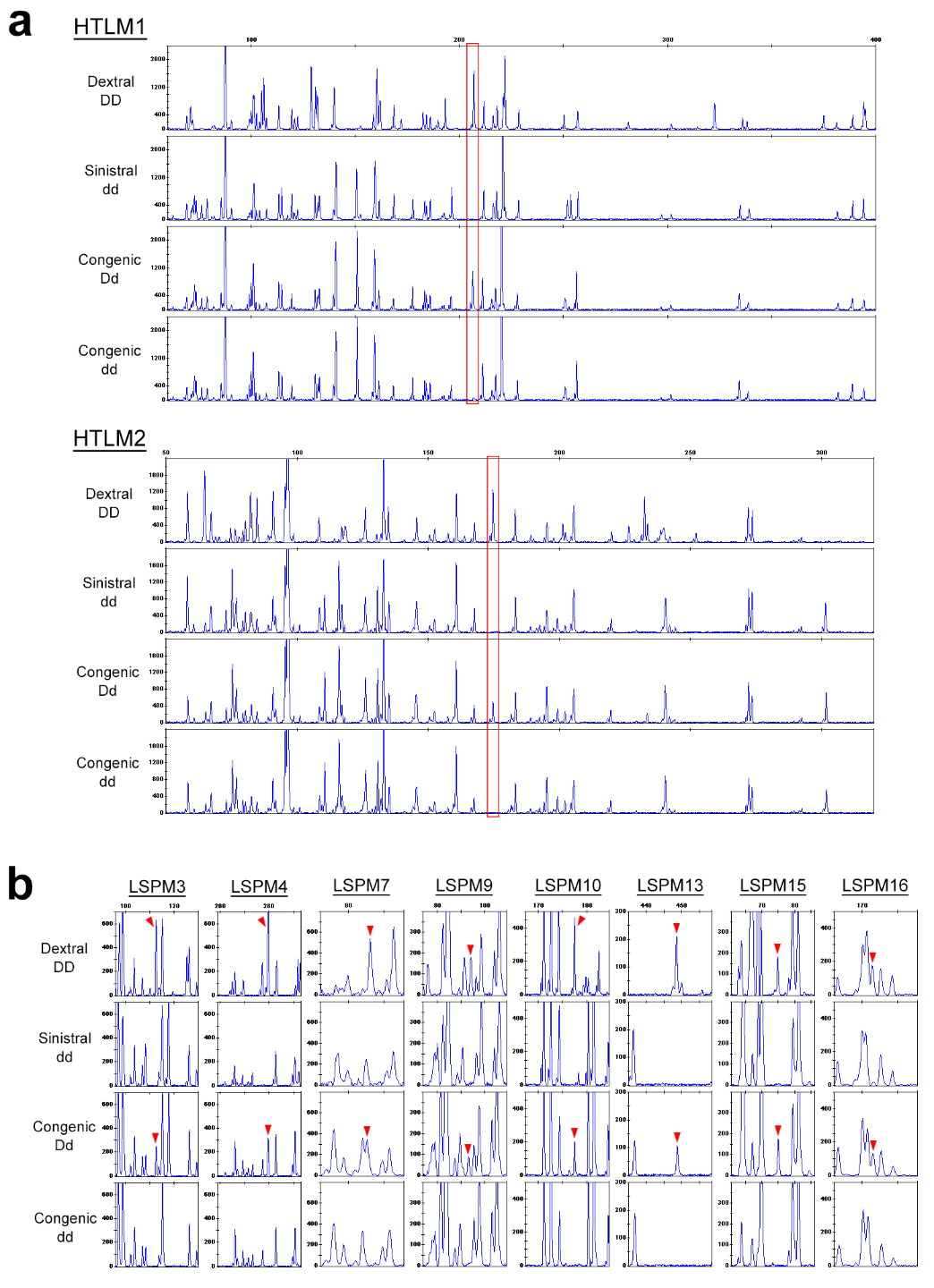


**Supplementary Figure S1: Detection of AFLP markers linked to the handedness-determining gene locus using a capillary electrophoresis system.**

Comparison of fragment patterns was performed using an Applied Biosystems 3130xl Genetic Analyzer and GeneMapper software. (a) Electropherograms show representative results of *Eco*RI/*Mse*I-AFLP typing for the detection of HTLM1 and HTLM2 markers. The peaks present in the red boxes show the linkage markers HTLM1 and HTLM2, which are linked to the dominant genotype for a handedness determining gene, DD or Dd. (b) Detection of linkage markers by *Pst*I/*Mse*I-AFLP typing are shown in the panels for each marker. Red arrowheads indicate the peaks corresponding to linkage marker fragments.


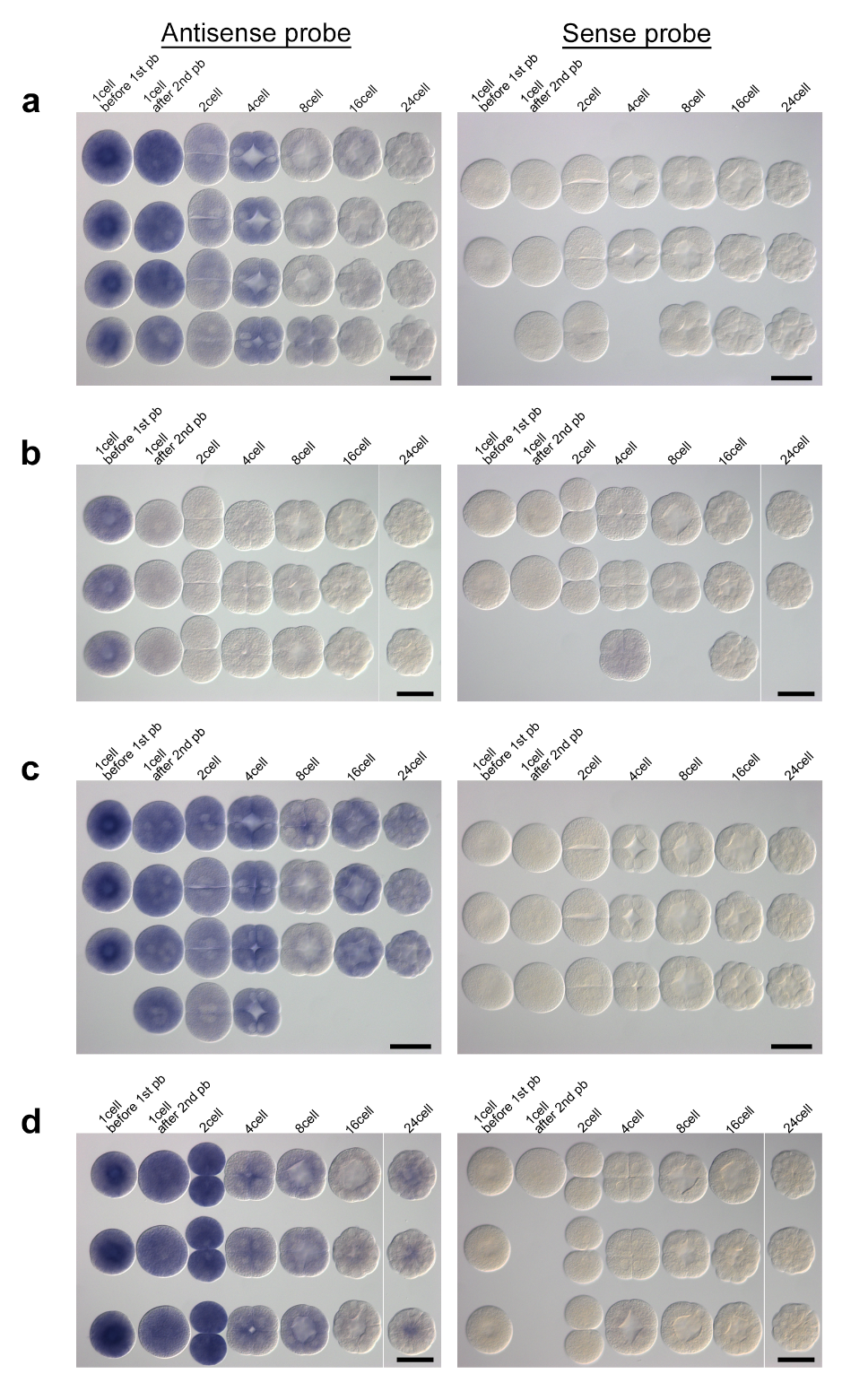


**Supplementary Figure S2: Distribution patterns of *Lsdia1* and *Lsdia2* mRNA in early cleaving embryos.** *Lsdia1* mRNA was detected by using LsDia1-N antisense probe in the early embryos of dextral (a) and sinistral (b) strains (left hand panels). *Lsdia2* mRNA was detected by using LsDia2-N antisense probe in the early embryos of dextral (c) and sinistral (d) strains (left hand panels). *In situ* hybridization of LsDia-N probe series were performed under the same conditions for the embryos of all stages for each probe. In addition, the staining time was the same for all samples (30°C, 8 hours). No signal was detected using the sense probe in each control experiment (right hand panels). Embryos of the same developmental stage were placed in a vertical column and aligned according to the developmental stages in one frame per probe for image capturing purposes. Scale bar 100 μm.


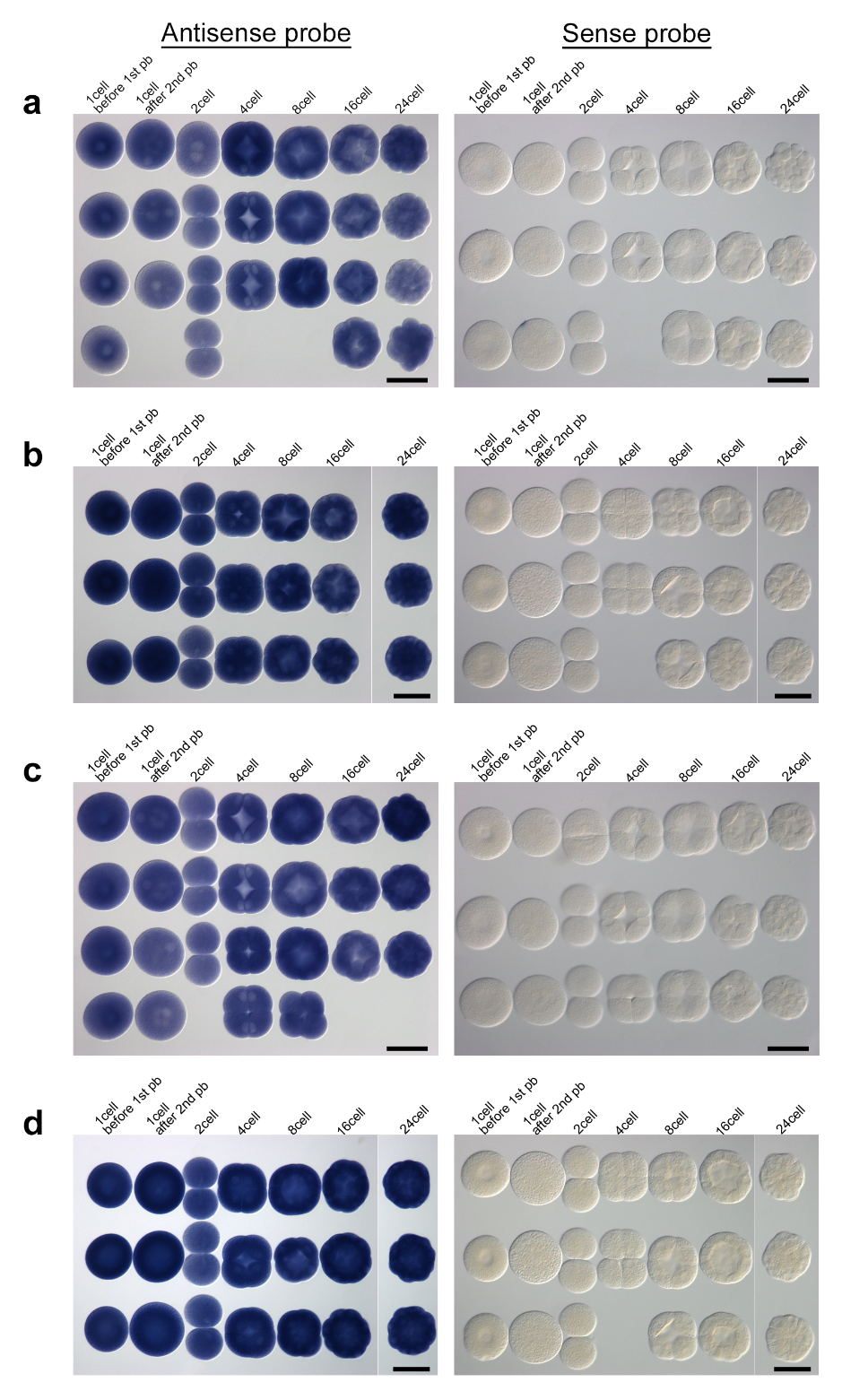


**Supplementary Figure S3: Distribution patterns of β-actin and β-tubulin mRNA in the early cleaving embryos as positive control.** *Lsactb1* mRNA was detected in the dextral (a) and sinistral (b) embryos, and *Lstubb1* mRNA was detected in the dextral (c) and sinistral (d) embryos by *in situ* hybridization using the respective antisense probes (left hand panels). *In situ* hybridization was performed under the same conditions for embryos of all stages for each probe. In addition, the staining time was fixed the same for each probe (RT, 48-180 minutes). No signals were detected by using the sense probe in each control experiment (right hand panels). Embryos of the same developmental stage were placed in a vertical column and aligned according to the developmental stages in one frame per probe for image capturing purposes. Scale bar 100 μm.


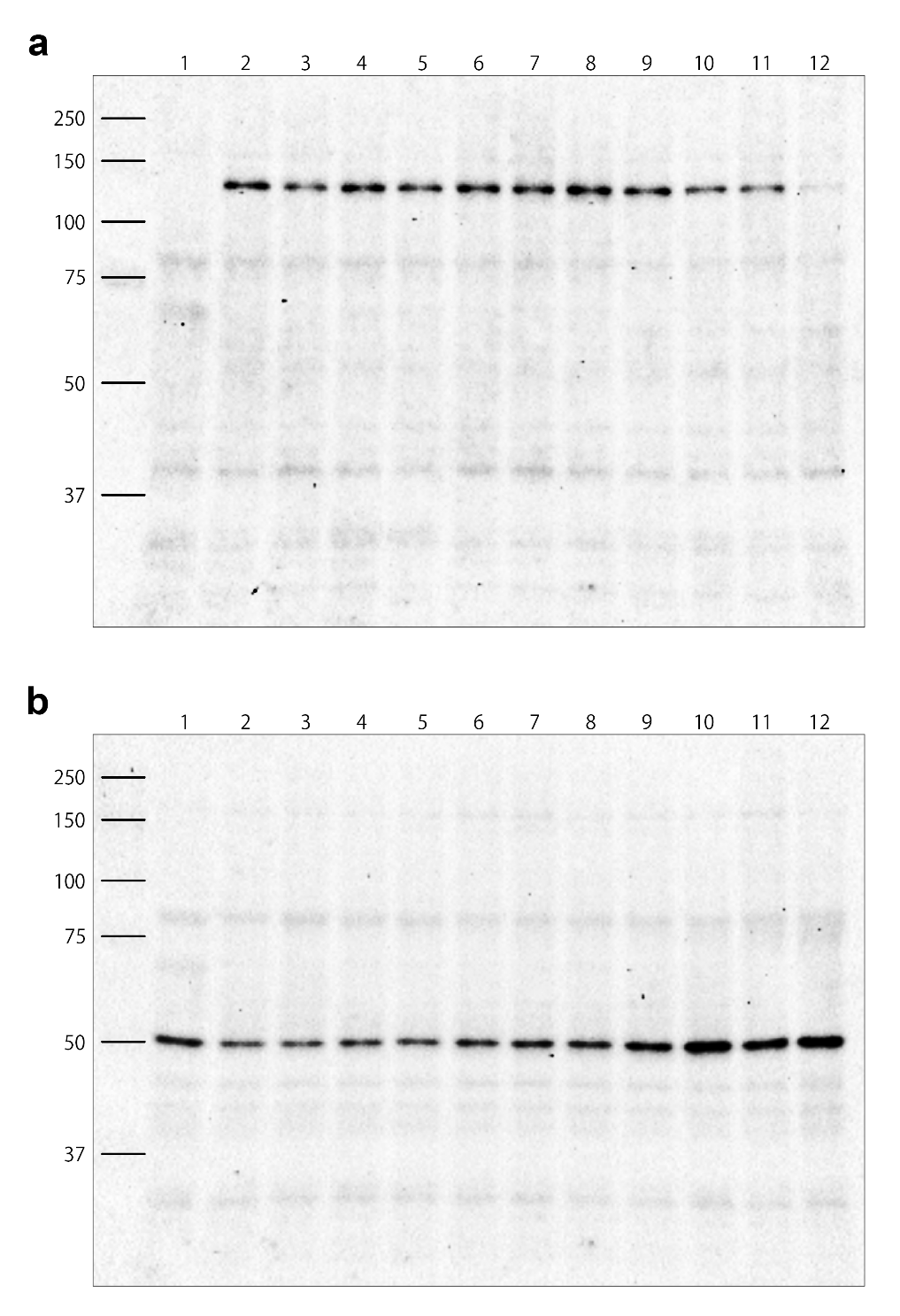


**Supplementary Figure S4: Western blot detection of LsDia1 protein in the early developmental stages of *L stagnalis.***

These images represent the whole gel images of Fig. 3b. Western blots were probed with anti-LsDia1 antibody (a) or anti-β-Tubulin antibody (b) using the same membrane. Positions of molecular weight marker (kDa) are indicated on the left side by CBB staining the membrane after western blotting. Lane 1; sinistral 1-cell before 1st pb extrusion. Lanes 2-12: dextral embryos. 2; 1-cell before 1st pb extrusion, 3;1-cell after 2nd pb extrusion, 4; 2-cell, 5; 4-cell, 6; 8-cell, 7; 12-16-cell, 8; 24-cell, 9; 49-64-cell, 10; Middle blastula, 11; Late blastula stages, 12; Gastrulating embryo. LsDia1 bands of the predicted molecular size (123.4 kDa), and β-Tubulin bands of the expected molecular size (50 kDa) are observed in (A) and (B), respectively.


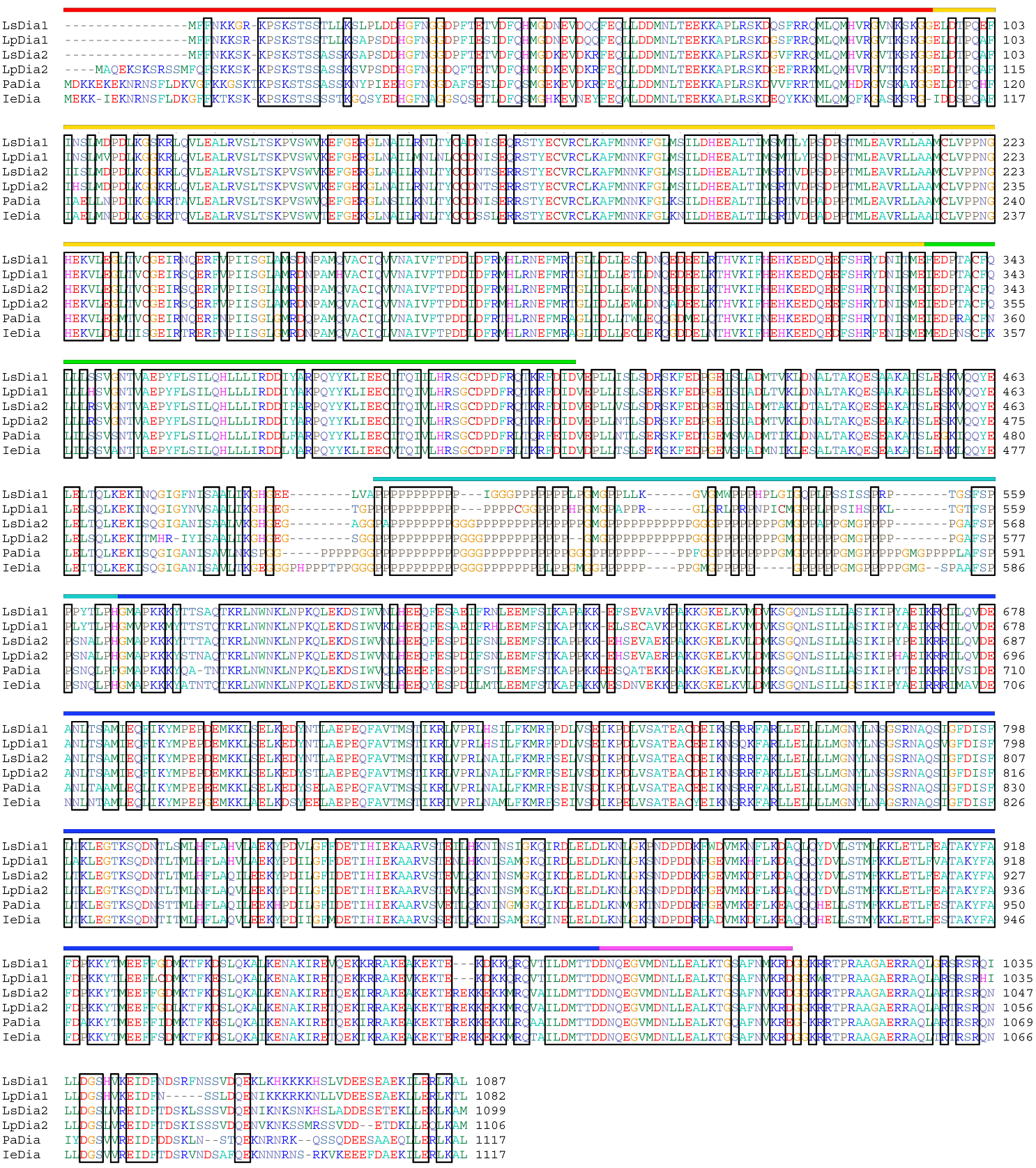


**Supplementary Figure S5: Alignment of Dia family proteins of pond snails.**

Amino acid sequences of Dia proteins of pond snails are very similar. Identical amino acids are boxed and the predicted domain structures are indicated by color lines, GBD (red), DID (yellow), DD (green), FH1 (light blue), FH2 (blue) and DAD (pink).

**Supplementary Table S1: *Eco*RI/*Mse*I-AFLP marker screening using a backcross panel to search for recombinants**

a: Genotype was judged from the offspring shell coiling.

b: Individuals, for which the genotype could not be identified by offspring shell coiling, were assigned from the pattern of AFLP markers.

c: Recombination occurred at either side of *Eco*RI/*Mse*I-AFLP marker against the genotype.

**Supplementary Table S2: AFLP marker typing of recombinants.**

The presence (1) or absence (0) of AFLP marker patterns are indicated. Marker positions denoting that meiotic recombination had occurred are shown in red. Region surrounded by a thick solid line indicates that LSPM 4, 10 and 15 are tightly linked to the handedness-determining gene locus.

a: Pure Dex-F4 and Pure Sin-F3 were parental individuals used as a source of backcross ADS line, and Pure Sin-F4 was the progeny of the sinistral line.

b: Dextral pure line (Dex); Sinistral pure line (Sin); Genotype indicated by the combination with each backcross generation and genotype (D or S). Genotype enclosed in brackets denotes presumptive genotype from the AFLP marker pattern.

**Supplementary Table S3: Fine mapping of recombination breakpoints.**

Fine mapping was performed by analysis of SNP patterns in the marker sequence. These types were classified as homozygous dextral (DD), sinistral (dd) or heterozygous (Dd) patterns. Marker positions for meiotic recombination are shown in red.

a: Pure Dex-F4 and Pure Sin-F3 were parental individuals used as a source of backcross ADS lines, and Pure Sin-F4 was the progeny of the sinistral line.

b: Dextral pure line (Dex); Sinistral pure line (Sin); Genotype indicated by the combination with the each backcross generation and genotype (D or S). Genotype enclosed in brackets means presumptive genotype from the AFLP marker pattern.
